# LRBA, a BEACH protein mutated in human immune deficiency, is widely expressed in epithelia, exocrine and endocrine glands, and neurons

**DOI:** 10.1038/s41598-024-60257-6

**Published:** 2024-05-09

**Authors:** Eleni Roussa, Pavel Juda, Michael Laue, Oliver Mai-Kolerus, Wolfgang Meyerhof, Markus Sjöblom, Katerina Nikolovska, Ursula Seidler, Manfred W. Kilimann

**Affiliations:** 1 Department Molecular Embryology, Institute of Anatomy and Cell Biology, Faculty of Medicine, Albert-Ludwigs-University Freiburg, Freiburg, Germany; 2 Department of Molecular Neurobiology, Max-Planck-Institute for Multidisciplinary Sciences, Göttingen, Germany; 3 Advanced Light and Electron Microscopy (ZBS 4), Robert Koch Institute, Berlin, Germany; 4 Department of Molecular Genetics, German Institute for Human Nutrition, Potsdam-Rehbruecke, Germany; 5 Department of Medical Cell Biology, Uppsala University, Uppsala, Sweden; 6 Department of Gastroenterology, Hepatology, Infectiology and Endocrinology, Medical University Hannover, Hannover, Germany; 7 Present Address: Leukocyte Motility Lab, 1st Faculty of Medicine, Charles University of Prague, Vestec, Czech Republic; 8 Present Address: Einstein Center for Neurosciences, Charite – Universitätsmedizin Berlin, Berlin, Germany; 9 Present Address: Center for Integrative Physiology and Molecular Medicine, Saarland University, Homburg, Germany

**Keywords:** Cell biology, Genetics, Immunology, Physiology, Systems biology, Anatomy, Diseases, Gastroenterology, Medical research, Molecular medicine

## Abstract

Mutations in LRBA, a BEACH domain protein, cause severe immune deficiency in humans. LRBA is expressed in many tissues and organs according to biochemical analysis, but little is known about its cellular and subcellular localization, and its deficiency phenotype outside the immune system. By LacZ histochemistry of *Lrba* gene-trap mice, we performed a comprehensive survey of LRBA expression in numerous tissues, detecting it in many if not all epithelia, in exocrine and endocrine cells, and in subpopulations of neurons. Immunofluorescence microscopy of the exocrine and endocrine pancreas, salivary glands, and intestinal segments, confirmed these patterns of cellular expression and provided information on the subcellular localizations of the LRBA protein. Immuno-electron microscopy demonstrated that in neurons and endocrine cells, which co-express LRBA and its closest relative, neurobeachin, both proteins display partial association with endomembranes in complementary, rather than overlapping, subcellular distributions. Prominent manifestations of human LRBA deficiency, such as inflammatory bowel disease or endocrinopathies, are believed to be primarily due to immune dysregulation. However, as essentially all affected tissues also express LRBA, it is possible that LRBA deficiency enhances their vulnerability and contributes to the pathogenesis.

## Introduction

BEACH proteins constitute a protein family of nine members in mammals, and are implicated in various aspects of membrane dynamics and membrane protein targeting^1^. They are large (~ 300 kDa), peripherally membrane-associating cytosolic proteins which seem to represent a novel but poorly understood molecular principle in the control of subcellular protein targeting.

Several BEACH proteins are involved in human diseases. Mutations of *LRBA*, in particular, give rise to severe immunodeficiency (hypogammaglobulinemia, recurrent infections) and immune dysregulation with a plethora of autoimmune manifestations^2^ (review^3^). LRBA deficiency causes T cell dysfunction and mistargeting of the immune receptor CTLA-4^4^, but other cell types of the immune system are also affected and may contribute e.g. to inflammatory bowel disease^5,6^. Though the clinical picture of human LRBA deficiency is dominated by the immunopathology, LRBA is expressed in many mammalian tissues according to transcript and Western blot analysis, highest in stomach and kidney^7^. LRBA-KO mice are viable and fertile, but do display immunological abnormalities^5,6,8–10^, and also olfactory and auditory impairments associated with mislocalization of the olfactory G-protein G_olf_ in olfactory sensory neurons^7^ and the adaptor proteins Radixin and Nherf2 in auditory hair cells^11^, respectively.

Cellular and subcellular localizations and functions of LRBA in tissues other than the aforementioned have remained unexplored. To identify candidate tissues and cell types whose functions might be affected by LRBA deficiency, we have carried out a survey of the histological pattern of LRBA expression in mouse tissues, taking advantage of a hypomorphic gene-trap mouse which expresses β-galactosidase activity under the control of the *Lrba* promoter. We then visualized, at higher resolution, LRBA protein expression and subcellular localization by immunofluorescence (IF) microscopy in several tissues of interest defined by the LacZ survey, particularly in the gastrointestinal tract, in exocrine and endocrine glands, and in taste buds. In select neurons and endocrine cells, this was complemented by immuno-electron microscopy (EM). Finally, given the widespread expression of LRBA in gastrointestinal epithelia, in exocrine glands, and in sensory epithelia, we investigated a number of gastrointestinal solute and water transport functions, as well as taste perception, for a possible impairment in LRBA-KO mice.

## Results

### LRBA is expressed in epithelia, exocrine and endocrine glands, and neuronal subpopulations

**Figure 1:**
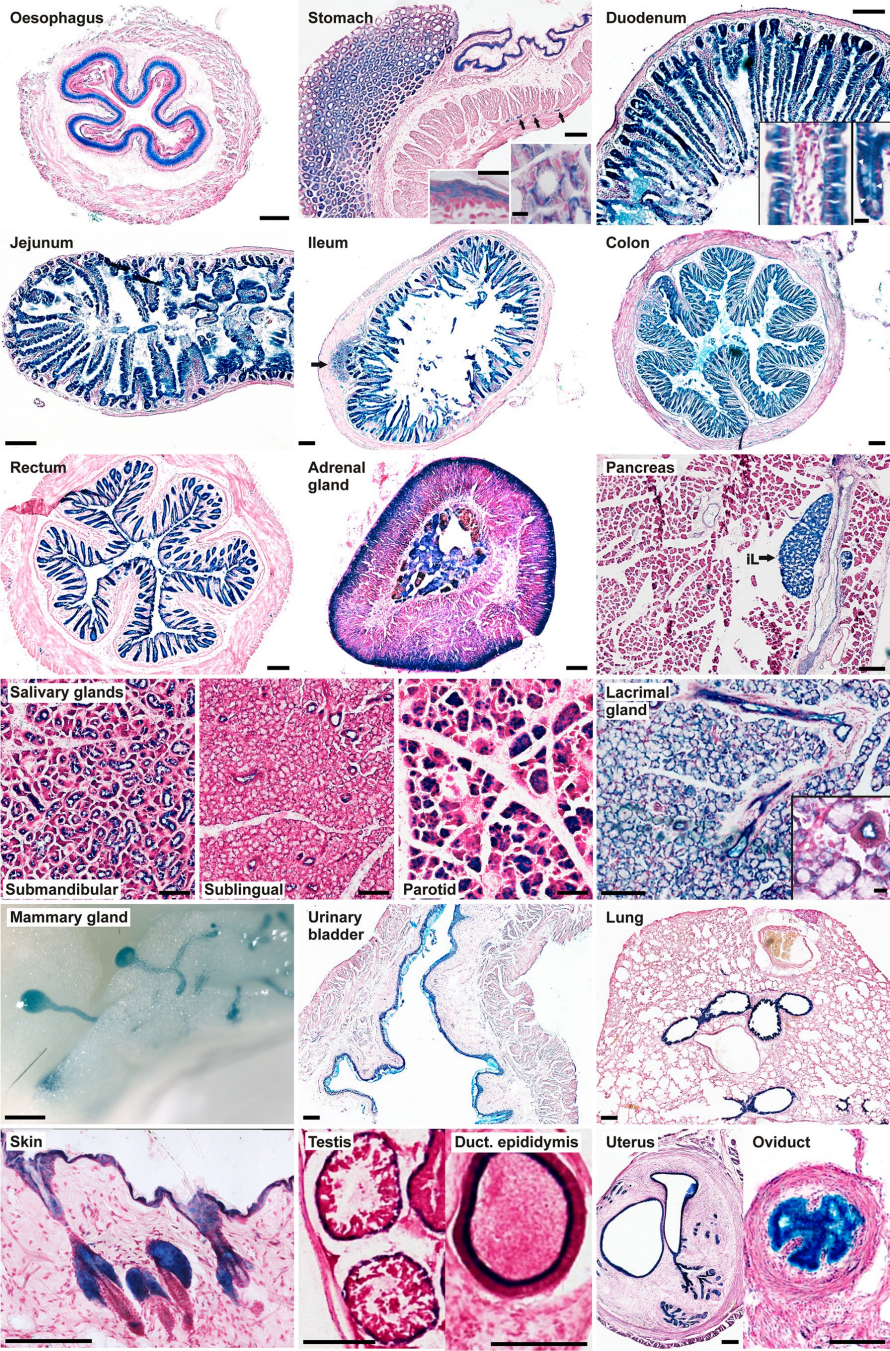
LacZ histochemistry detects widespread expression of *Lrba* in epithelia, exocrine and endocrine glands. Most images were recorded from cryosections stained by LacZ histochemistry, except oesophagus (paraffin section of a whole-mount staining) and mammary gland (stereomicroscopy of a whole-mount staining). (Stomach) Left inset: squamous epithelium of the forestomach; right inset: cross-sectioned crypts of the glandular stomach; arrows: cross-sectioned fibers of the myenteric plexus. (Duodenum) Left inset: close-up of a longitudinally sectioned villus; right inset: close-up of a longitudinally sectioned crypt (white arrowheads indicate mucus-filled goblet cells). (Ileum) Arrow indicates a lymph follicle in the ileum wall. (Pancreas) *iL* islet of Langerhans. (Lacrimal gland) Inset shows close-up with cross-sectioned duct. Scale bars: 100 µm in main frames, 10 µm in insets.

The *Lrba/gt* gene-trap mouse (official designation, *Lrba*^*Gt(XE315)Byg*^^7^), is hypomorphic for endogenous Lrba (~ 25% residual expression^7^) and also expresses β-galactosidase (LacZ), fused to the N-terminal 71% of the endogenous LRBA coding sequence, under the control of the *Lrba* gene promoter. This enabled a comprehensive survey of the mouse tissues and cell types in which *Lrba* is expressed, by LacZ reporter histochemistry. Through LacZ staining of tissue sections or whole-mount preparations (Fig. 1), we detected expression in all epithelia investigated, as well as in the mesothelial ependyma and peritoneum. Marked staining was also observed in several secretory epithelial appendages of the gastrointestinal tract and the skin. Endocrine cells of the adrenal gland and pancreas, and multiple neuron populations in the brain and spinal cord were also stained. LacZ staining of wild-type (WT) control specimens was always carried out in parallel, and in all instances shown, control staining was absent or much fainter. Some tissues could not be evaluated because of high WT background hydrolase staining, such as bone, cartilage, ovary, and accessory glands of the male reproductive tract. Specific LacZ staining was notably absent in skeletal, cardiac and smooth muscles, and in adipocytes.

Beginning from top-left, Fig. 1 shows LacZ staining of the squamous epithelia of the oesophagus and of the fore-stomach (right half of the stomach image, and left inset). Cells of the glandular stomach (left half of the stomach image) displayed a mosaic of stronger and weaker staining at higher resolution (right inset), presumably reflecting the different cell types of the gastric mucosa. Staining of the peritoneum and of the cross-sectioned myenteric plexus (arrows) was also seen. Throughout the intestine, the mucosal epithelia of the duodenum, jejunum, ileum, colon and rectum were LacZ-positive, and most markedly the crypts (duodenum, right inset) at the bases of villi in the duodenum, jejunum and ileum, whereas interstitium (duodenum, left inset) and smooth muscle remained faintly stained or unstained, respectively. LacZ-positive lymph follicles, as in the ileum (arrow), were often seen. In a section of the adrenal gland, the endocrine cells of the medulla and of the cortical zona glomerulosa stand out as LacZ-positive. In the pancreas, the endocrine Langerhans islets are strongly stained (iL), whereas less marked LacZ staining is seen in the ducts and acini of the exocrine pancreas. In all salivary glands, ducts are stained, whereas acini are LacZ-positive only in the submandibular and parotid, but not in the sublingual gland. The lacrimal gland displays pronounced staining of ducts as well as acini. In a whole-mount of virginal mammary gland, LacZ-positive ducts and acinar buds stand out against the surrounding unstained adipose tissue. The epithelium of the urinary bladder is also LacZ-positive. In the lung, the bronchiolar epithelium is selectively stained whereas the alveolar parenchyma appears LacZ-negative. The epidermis of the skin surface and of the hair shafts is LacZ-positive, and particularly so the sebacious glands flanking the hair follicles. In the seminiferous tubules of the testis, LacZ staining is limited to the peripheral, monocellular layer of spermatogonia whereas their progeny cell types and the Sertoli cells towards the lumen are unstained. The epithelium of the epididymal duct is specifically stained while accessory glands of the male reproductive tract could not be evaluated because of high β-galactosidase background staining also in the WT tissues. In the female reproductive tract, the endometrium, uterine glands and oviduct epithelium showed marked LacZ staining, whereas in the ovaries (not shown) slight but specific staining was seen in the corpora lutea while the stroma displayed high unspecific staining also in the WT control. LacZ staining was also observed in hepatocytes and in the epithelium of the gallbladder (not shown).

**Figure 2:**
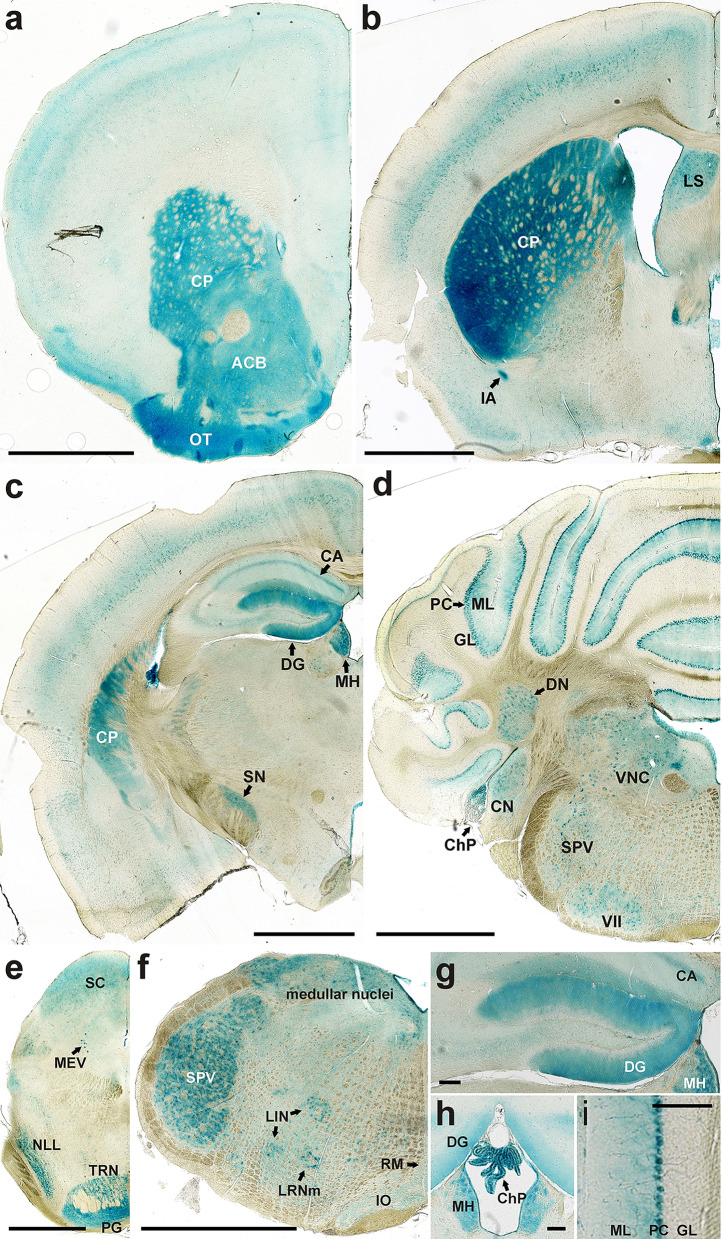
LacZ histochemistry detects differential *Lrba* expression levels in multiple neuronal cell populations. Frontal sections of the septo-striatal region (**a**), the septo-diencephalic region (**b**), the caudal diencephalon (**c**), the rostral cerebellum (**d**), the caudal mesencephalon (**e**) and the medulla oblongata (**f**) are shown in rostro-caudal order. The dentate gyrus (**g**), the 3rd ventricle with the chorioid plexus (**h**), and the cerebellar cortex (**i**) are shown at higher magnifications. Abbreviations of LacZ-positive features: *ACB* nucleus accumbens, *CA1* hippocampus (cornu ammonis), *ChP* chorioid plexus, *CN* cochlear nuclei, *CP* caudato-putamen, *DG* dentate gyrus, *DN* dentate nucleus, *GL* granular layer, *IA* intercalated amygdalar nucleus, *IO* inferior olivary complex, *LIN* linear nucleus of the medulla, *LRNm* lateral reticular nucleus magnocellular part, *LS* lateral septal nucleus, *MEV* midbrain trigeminal nucleus, *MH* medial habenula, *ML* molecular layer, *NLL* nucleus of the lateral lemniscus, *OT* olfactory tubercle, *PC* Purkinje cells, *PG* pontine gray, *RM* nucleus raphe magnus, *SC* superior colliculus, *SN* substantia nigra, *SPV* spinal nucleus of the trigeminal, *TRN* trigeminal reticular nucleus, *VII* facial motor nucleus, *VNC* vestibular nuclei. Scale bars: 1 mm in (**a–f**), 100 µm in (**g–i**).

In the central nervous system, staining intensities differed greatly between regions and cell populations. Figure 2a–f shows a series of frontal sections of the mouse brain along the rostral-caudal axis. The most intensely stained brain structures were the olfactory bulb (see^7^), the caudato-putamen (CP; Fig. 2a–c), the dentate gyrus (DG; Fig. 2c,g), the Purkinje cells (PC; Fig. 2d,i) of the cerebellum, and the ependyma, most strikingly at the chorioid plexus (ChP; Fig. 2h). The dentate gyrus (Fig. 2g), the third ventricle with its LacZ-positive ependyma lining and chorioid plexus (Fig. 2h), and the cerebellar cortex with the granule cell (GL), Purkinje cell (PC) and molecular (ML) layers (Fig. 2i) are therefore also shown at higher magnifications. Other LacZ-positive features are identified in the legend to Fig. 2. Whereas individual LacZ-positive neuronal cell bodies stand out in various brain-stem nuclei and the Purkinje cell layer (Fig. 2d–f,i), LacZ label in other regions such as the caudato-putamen (CP; Fig. 2a–c), the olfactory tubercle (OT; Fig. 2a) or the pontine grey (PG; Fig. 2e) appears diffuse and is probably localized to the neuropil. Similarly, the diffuse LacZ staining of the stratum moleculare of the dentate gyrus (DG; Fig. 2c,g,h) probably reflects the dendritic trees of the DG granule cells. As the LRBA-LacZ fusion protein contains only 71% of the LRBA amino acid (a.a.) sequence, it is unknown to what extent this partial sequence of LRBA contributes to the subcellular localization of the LacZ enzyme moiety.

**Figure 3:**
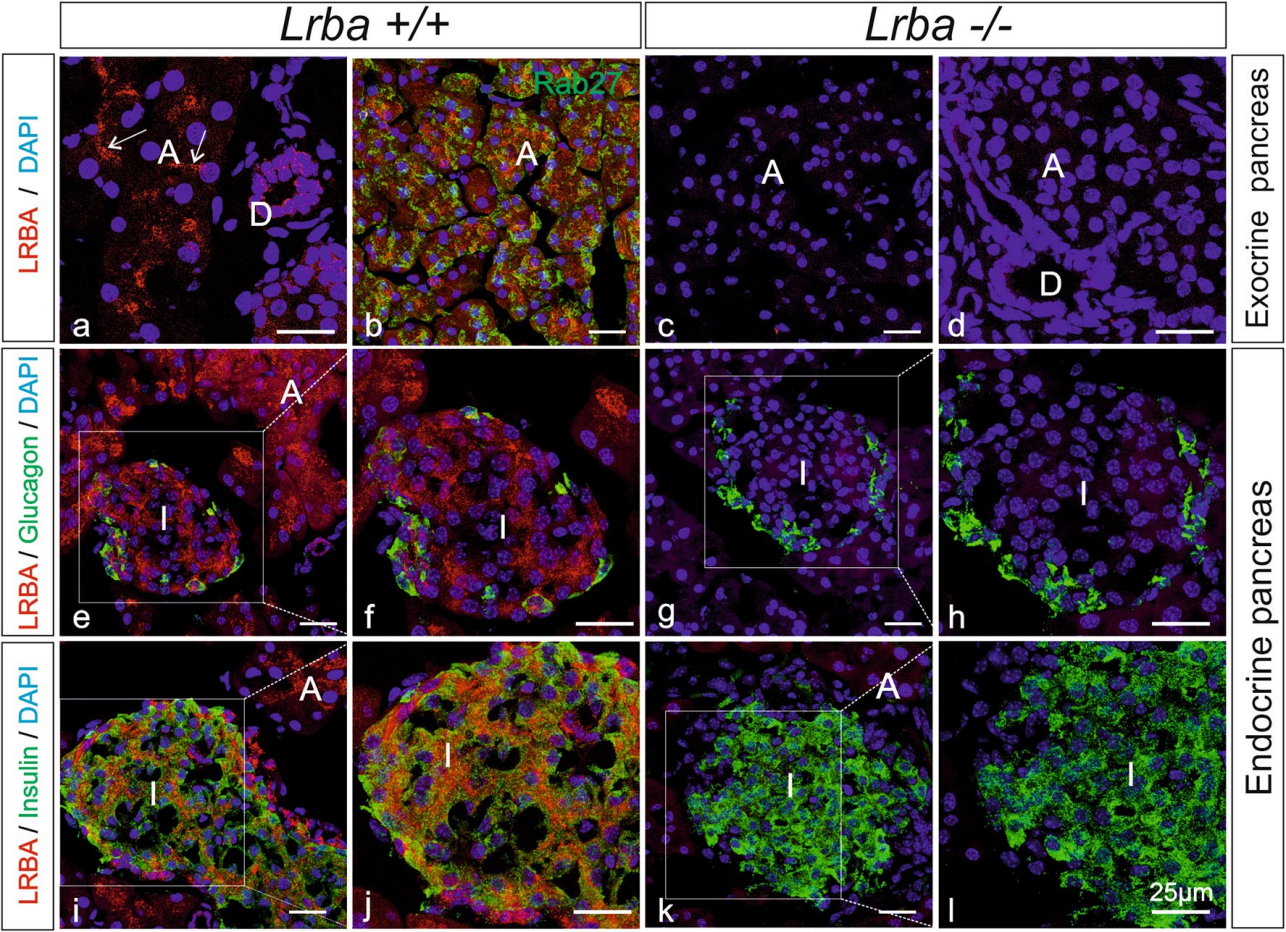
LRBA immunofluorescence in the exocrine and endocrine pancreas. Double-immunofluorescence confocal microscopy for LRBA (red) and for either Rab27 (**b**), or glucagon (**e–h**) or insulin (**i–l**) (in green) in *Lrba*^+*/*+^ (**a,b,e,f,i,j**) and *Lrba*^−/−^ (**c,d,g,h,k,l**) mice. Nuclei were stained with DAPI (blue). (**f,h,j,l**) Represent higher magnification pictures of the white-boxed areas in (**e,g,i,k**), respectively. Arrows in (**a**) point to LRBA label concentrating toward the apical poles of acinar and intercalated duct cells. LRBA labelling was not detectable in LRBA-KO mouse tissue. *A* acini, *D* ducts, *I* islets. All scale bars: 25 µm.

**Figure 4:**
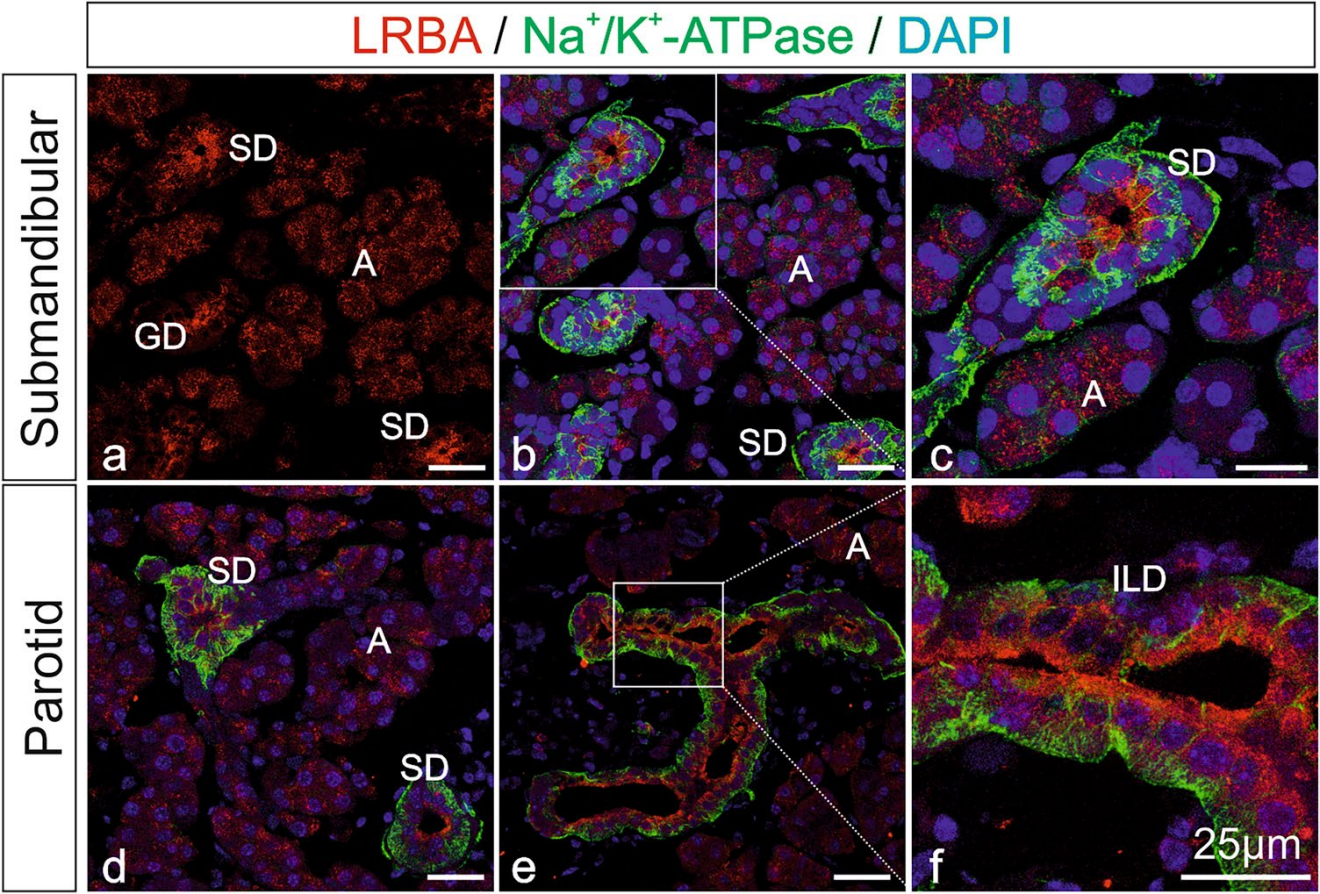
LRBA immunofluorescence in salivary glands. Double-immunofluorescence confocal microscopy on mouse submandibular and parotid gland for LRBA (red) and for Na^+^/K^+^-ATPase (green) as a marker of basolateral plasma membranes. Nuclei were stained with DAPI. (**c,f**) Represent higher magnification images from the white-boxed areas in (**b,e**), respectively. LRBA immunofluorescence concentrated toward the subapical and apical cytoplasm of acinar and duct cells, and was abolished in KO control tissue (Supplementary Fig. S1b). *A* acinar cells, *GD* granular duct, *ILD* intralobular duct, *SD* striated duct. All scale bars: 25 µm.

**Figure 5:**
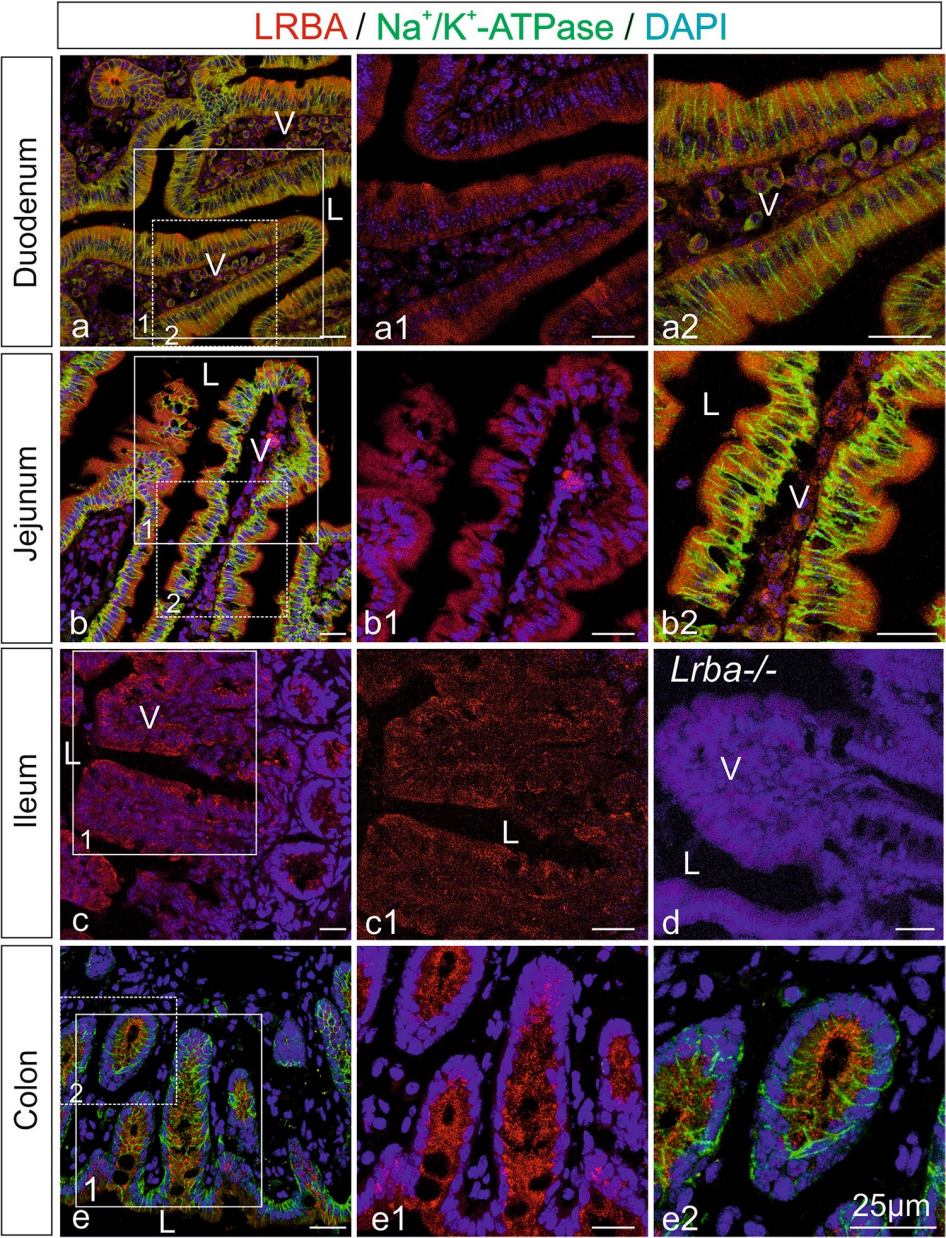
LRBA immunofluorescence in intestinal epithelia. Single LRBA immunofluorescence (red; **a1,b1,c,c1,e1**) and double immunofluorescence with Na^+^/K^+^-ATPase (green; **a,a2,b,b2,e,e2**), a marker for basolateral plasma membranes, and subsequent confocal microscopy on mouse intestine. Nuclei were stained with DAPI. (**a1,a2,b1,b2,c1,e1,e2**) Represent higher magnification images from the white-boxed areas in (**a–c,e**). In jejunum and colon, LRBA immunofluorescence concentrated at the apical cell poles and subapical regions of epithelial cells. In LRBA-KO tissue, LRBA IF was absent (**d**). *V* intestinal villi, *L* lumen. All scale bars: 25 µm.

To confirm the LacZ findings in selected tissues with an independent technique, and to determine with higher resolution in which cell types and subcellular regions the LRBA protein is localized, we next performed immunofluorescence (IF) microscopy (Figs. 3, 4, 5), always staining LRBA-KO mouse tissues in parallel as specificity controls (*Lrba*^*tm1.1Kili*^)^7^.

In the exocrine pancreas (Fig. 3), Lrba was detected in both acinar (A) and duct (D) cells (Fig. 3a,b). The subcellular distribution of LRBA IF in acinar cells was reminiscent of zymogen granule clusters, but double immunofluorescence for LRBA and the zymogen granule markers Rab27 (Fig. 3b and at higher magnification in Supplementary Fig. S1a, green) and amylase (not shown) indicated no co-localization of LRBA and zymogen granules at confocal resolution. LRBA appeared to concentrate toward the lumenal poles of centroacinar cells and intercalated ducts (Fig. 3a, arrows). Also in the epithelial cells of intralobular ducts (D), LRBA concentrated in the cytoplasm below the apical (lumenal) plasma membrane. In *Lrba*^−/−^ mice (Fig. 3c,d), LRBA IF in both acinar and duct cells was abolished. LRBA IF was also seen in endocrine Langerhans islets (I). Double-IF of LRBA with either glucagon (Fig. 3e,f) or insulin (Fig. 3i,j) indicated LRBA expression in both glucagon-positive α cells and insulin-positive β cells (Fig. 3j). In *Lrba*^−/−^ mice no LRBA was detectable in islets, while the expression and subcellular distribution of glucagon (Fig. 3e–h) and insulin (Fig. 3i–l) appeared unaffected.

In WT mouse salivary glands (Fig. 4), LRBA IF was seen in both acinar (A) and duct cells of the submandibular (4a–c) and parotid glands (4d–f), similar to the exocrine pancreas. In acinar cells, LRBA IF was distributed throughout the cytoplasm in a granular or patchy pattern. In all duct segments, i.e. granular ducts (GD), striated ducts (SD) and intralobular ducts (ILD), LRBA label was more intense than in acini and markedly shifted toward the apical cell pole. Double-IF for Na^+^/K^+^-ATPase (green) was chosen to highlight the basolateral plasma membranes specifically of duct cells (Fig. 4b–f). In *Lrba*^-/-^ mouse control tissues, LRBA IF was absent (Supplementary Fig. S1b).

Figure 5 shows LRBA IF in intestinal epithelia [duodenum (a,a1,a2), jejunum (b,b1,b2), ileum (c,c1) and colon (e,e1,e2)], in accordance with the LacZ staining of Fig. 1. In all tissues in which both, specific LacZ and IF staining were performed, concordant staining patterns of cell types were seen. In enterocytes of the prominent villi (V) of duodenum, LRBA IF was weak and diffusely intracellularly distributed without polarization (Fig. 5a,a1,a2). In the villi of jejunum (Fig. 5b,b1,b2), LRBA IF concentrated in the apical and subapical regions of the enterocyte cytoplasm, opposite to the localization of Na^+^/K^+^-ATPase (green) at the basolateral plasma membranes. Also in ileum (Fig. 5c,c1), LRBA IF tended toward the luminal enterocyte cell poles, but was undetectable in tissue from *Lrba*^−/−^ mice (Fig. 5d). In the crypts of colon (Fig. 5e, e1,e2), LRBA IF labeling was again predominant at the luminal cell side of the colonocytes, opposite to the polarity of the Na^+^/K^+^-ATPase.

### Differential subcellular distributions of LRBA and NBEA within neurons and endocrine cells

The closest relative of LRBA within the BEACH protein family is Neurobeachin (NBEA). Both proteins are co-expressed in neurons and endocrine cells, and GTP stimulates both to associate with a *trans*-Golgi-near endomembrane compartment in permeabilised cells in vitro^7,12^. We therefore investigated by immunogold electron microscopy (EM) whether, in cell types which co-express LRBA and NBEA, their steady-state subcellular localizations are similar or distinct.

**Figure 6:**
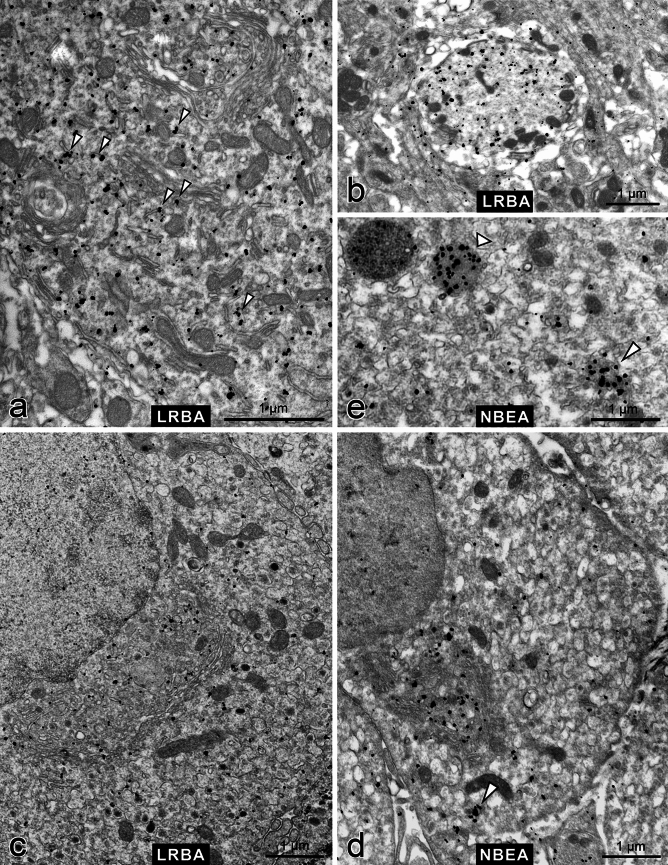
Immuno electron microscopy demonstrates differential subcellular distributions of LRBA and NBEA. Pre-embedding immunolabelling with anti-LRBA (**a–c**) or anti-NBEA (**d,e**) was followed by nanogold-conjugated secondary antibody and silver-enhancement. (**a**) Purkinje cell cytoplasm with two Golgi complexes (top and center) and occasional association of LRBA immunolabel with endomembranes (arrowheads). See also Supplementary Fig. S2c. (**b**) Cross-sectioned Purkinje cell dendrite (LRBA-positive) surrounded by the largely LRBA-negative neuropil of the molecular layer of the cerebellum. (**c**) Adrenal chromaffin cell with a typical, convoluted paranuclear Golgi complex; LRBA immunolabel is scattered throughout the cytoplasm, slightly enriched near the concave face of the Golgi complex. See also Supplementary Fig. S2d. (**d**) Adrenal chromaffin cell; NBEA immunolabel is strikingly enriched in the concave space of the Golgi complex. See also Supplementary Fig. S2e. (**e**) Close-up of a chromaffin cell shows NBEA immunolabel decorating the lumen of a lysosome-like organelle (broad arrowhead) whereas another putative lysosome to the left is NBEA-negative; and an aggregate of small vesicles and diffuse material (slim arrowhead). Such an aggregate is also pointed out by an arrowhead in part (**d**). See also Supplementary Fig. S2f.

In Purkinje cells and other neurons, NBEA is strikingly associated with vesiculo-tubulo-cisternal endomembrane profiles, concentrating at the concave faces and margins of Golgi stacks^12^ (Supplementary Fig. S2a). In contrast, LRBA immunolabel was seen scattered throughout the Purkinje cell cytoplasm (Fig. 6a) and extending into dendrites (Fig. 6b), with occasional association with endomembranes (arrowheads) but no enrichment near Golgi complexes (see a curved Golgi stack at the top and a flat stack in the middle of Fig. 6a). Virtually no labelling was seen in specimens from LRBA-KO mice processed in parallel (Supplementary Fig. S2b). Further documentation of LRBA immunodecoration specificity can be seen in Supplementary Fig. S2c, which again shows strong, evenly distributed labelling of Purkinje cell cytoplasm while neighbouring Golgi cells are unlabelled, consistent with the Purkinje cell-specific LRBA expression in cerebellum shown by LacZ histochemistry in Fig. 2i.

Also in endocrine chromaffin cells, we observed this differential subcellular localization of LRBA (Fig. 6c) and NBEA (Fig. 6d): LRBA label was scattered more broadly over the cytoplasm (though slightly enriched near the Golgi complex) and was often associated with small vesicles (Supplementary Fig. S2d), whereas NBEA strikingly concentrated *trans*-Golgi-near and tended to be associated with larger tubulo-cisternal membrane profiles (Fig. 6d, Supplementary Fig. S2e). In chromaffin cells, we saw NBEA immunolabel also decorating the lumen of a subset of lysosome-like organelles (Fig. 6e, broad arrowhead), and aggregates of small vesicles and amorphous electron-dense material (slim arrowhead; see also Supplementary Fig. S2f).

### LRBA is expressed in taste sensory epithelial cell types, but LRBA-KO mice perform normally in gustatory tests

Our attention was attracted to taste perception, because (1) whole-mount LacZ histochemistry stained the groove of the vallate papilla at the base of the mouse tongue (not shown), and because (2) the gustatory G-protein (Gustducin) shares the γ subunit isoform Gng13 with the olfactory G-protein, G_olf_, whose functional expression is impaired in LRBA-KO mice^7^. The shared Gng13 subunit might be critical for the functional expression of both heterotrimeric GTPases. We therefore investigated whether LRBA is expressed in taste chemosensory cells, and whether the LRBA-KO affects Gustducin expression or taste perception.

**Figure 7:**
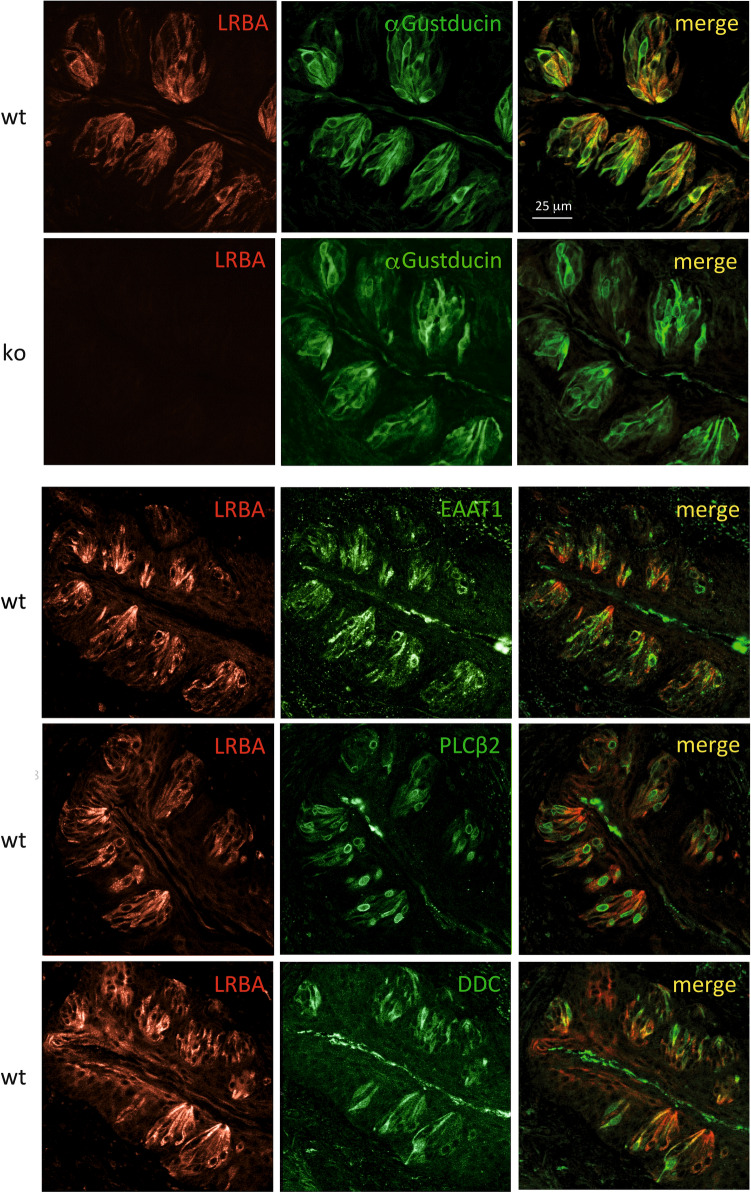
LRBA immunofluorescence in type I–III taste cells of vallate papilla taste buds. LRBA-IF-positive cells (left column) display high degrees of overlap with cells IF-positive for α-Gustducin, EAAT1 (type I cell marker), PLCβ2 (type II cell marker) and DDC (type III cell marker). In taste buds from LRBA-KO mice, LRBA-IF was not detectable, while the staining patterns of α-Gustducin (second row from top) or the other markers (not shown) seemed unaffected by the LRBA-KO.

IF microscopy detected LRBA in the vallate and foliate chemosensory papillae, and also in the filiform mechanosensory papillae (not shown). In the onion-shaped taste buds of the vallate papillae (Fig. 7), LRBA-IF stood out against the surrounding squamous epithelium which stained much fainter. Taste buds comprise three mature sensory cell types: type I “glial-like” cells express the excitatory amino acid transporter-1 (EAAT1); type II “receptor” cells confer sweet, bitter or umami taste, employing Gustducin and phospholipase C-β2 as signalling proteins; sour taste perception is ascribed to type III “presynaptic” cells^13,14^. Marked overlap of cells expressing LRBA was seen with α-Gustducin (expressed in type II cells) as well as with additional markers for all three cell types of the taste buds: type I (marker: EAAT1), type II (phospholipase C-β2 [PLCβ2]) and type III (Dopa decarboxylase [DDC]). Quantitative evaluation indicated that of 29 EEAT1-positive cells, 28 (97%) were also LRBA-positive; of 75 PLCβ2-positive cells, 61 (81%) were also LRBA-positive; and of 49 DCC-positive cells, 29 (59%) were also LRBA-positive. Therefore, LRBA is expressed in all three taste cell populations, most prominently in type I cells. LRBA staining was abolished in KO mouse vallate papillae (shown for example in Fig. 7 by double-IF with α-Gustducin), confirming specificity. Taste cells stained for α-Gustducin and the other cell type markers seemed unaffected by the LRBA-KO in number, appearance, or marker IF intensities.

Comprehensive lickometer tests^15^ were performed to investigate whether the LRBA-KO affected any of the five taste modalities: salty (tested with NaCl and KCl as tastants), sour (HCl, citric acid), sweet (sucrose, polycose, aspartam, sucralose), bitter (quinine, papaverin, cycloheximide, denatonium benzoate), and umami (sodium glutamate, potassium glutamate). We analysed three separate animal cohorts with a total of 22 KO and 21 WT mice (cf. Supplementary Fig. S3, Supplementary Table S1), but could not detect significant differences in attractive or aversive responses between the genotypes (data not shown).

### LRBA-KO mice displayed no detectable impairments of several transport functions in stomach, duodenum and jejunum

The high expression of LRBA in many exocrine glands and gastrointestinal epithelia prompted us to explore whether solute or water transport functions might be affected in LRBA-KO mice, in ways consistent with the cellular expression patterns of LRBA. Identification of physiological dysfunctions could lead to the identification of effector/cargo proteins whose proper subcellular localization requires LRBA.

As duodenal epithelial transport functions, we determined the mucosal bicarbonate secretion, mucosal paracellular permeability and mucosal net fluid flux in mice where the duodenal segment was perfused in vivo (Supplementary Fig. S4a–c). The basal rates of these parameters were stable during perfusion with isotonic saline over 30 min, and indiscriminable between control animals and LRBA-KO mice. Perfusing the duodenal segment with 1.0 µM of PGE_2_ during 20 min increased the rate of duodenal bicarbonate secretion in control animals and LRBA-KO mice alike (a). No differences in basal or PGE_2_-treated blood-to-lumen clearance of ^51^Cr-EDTA (b) or in mucosal net fluid flux (c) were observed.

To assess further epithelial functions of the upper and lower gastrointestinal tract, we tested LRBA-KO and WT littermates for: (a) Duodenal bicarbonate secretory rates (basal and acid-stimulated) in vivo (Supplementary Fig. S5a): The stimulation of duodenal alkaline output after a brief contact of the mucosa with a high proton concentration (but not high enough to cause epithelial damage: 3 × 10^−3^ M, 5 min) involves stimulation of neural circuits^16,17^, and can therefore only be tested in vivo. The rate of increase of the HCO_3_^−^ secretory rate after acid contact tended to be faster in the LRBA-KO duodenum, but neither the basal rate nor the stimulated rate 1 h after the acid contact were significantly different. (b) Basal and maximal acid secretory rates in isolated gastric mucosa: Supplementary Fig. S5b displays the acid secretory rates over time, basal and after addition of forskolin plus the phosphodiesterase inhibitor IBMX (10^−5^ M forskolin and 10^−4^ M IBMX). No significant difference was found between the two groups. (c) Fluid absorptive rate during perfusion of the jejunum with various luminal solutions, which is a functional measure for a complex array of ion and nutrient transporters^18–20^: The jejunal loop was sequentially perfused with an unbuffered sodium chloride solution, a CO_2_/HCO_3_^−^ buffered solution, and a glucose-containing CO_2_/HCO_3_^−^ buffered solution, and both the fluid absorptive rate and the pH of the outflowing perfusate were assessed. The basal fluid absorptive rate, upon perfusion with unbuffered saline, allows to estimate alkaline/acid output by the intestinal segment. The change to a CO_2_/HCO_3_^−^ containing perfusate stimulates fluid absorption via an enhanced activity of the apical Na^+^/H^+^ exchanger NHE3, which is stimulated by CO_2_ entering from the luminal bath^19,20^. The addition of glucose stimulates the apical Na^+^/glucose cotransporter SGLT1, but also activates the Na^+^/H^+^ exchanger NHE3^21^ as well as the Cl^−^/HCO_3_^−^ transporter Slc26a6^18^, involving insertion of these transporters into the plasma membrane from intracellular pools. As seen in Supplementary Fig. S5c, the switch to a CO_2_/HCO_3_^−^ buffered solution resulted in a mild stimulation, and the addition of glucose to the perfusate resulted in a strong stimulation of fluid absorption in both genotypes. However, there were no significant differences between the LRBA-KO and WT littermates regarding the fluid absorptive rates, or the pH of the outflowing solution.

## Discussion

Bi-allelic mutations in the human *LRBA* gene cause severe immune deficiency, affecting several cell types of the immune system and leading to the mis-trafficking of CTLA-4 and other immune receptors^4–6^. Therefore, the function of LRBA in the immune system is intensely investigated. In LRBA-KO mice, the impairments of immune functions are clinically milder than in human patients^5,8–10^. However, biochemical data indicate LRBA expression in essentially all tissues, but it was unclear which cell types express LRBA and what its functions outside the immune system might be.

In the present study, we employed the *Lrba/gt* gene-trap mice, which express β-galactosidase under the control of the *Lrba* promoter, to survey the histological pattern of *Lrba* expression in multiple mouse tissues by LacZ histochemistry. We detected expression in all epithelia, mesothelia, exocrine and endocrine cells investigated, though some tissues were uninformative due to a high background of endogenous β-galactosidase staining. In the brain and spinal cord, regions and neuronal cell types differed markedly in *Lrba* expression levels. Therefore, in epithelia and endocrine cells LRBA may be primarily involved in housekeeping functions, whereas in neurons its functions may be more cell-type specific. Muscle cells (smooth or striated) and adipocytes did not express *Lrba*.

LRBA is known to be critical for the subcellular trafficking of the immune receptor CTLA-4^4^ and of the olfactory G-protein G_olf_^7^, while its close relative, Neurobeachin (NBEA), is involved in the targeting of multiple ionotropic neurotransmitter receptors and of connexins^22–24^. We suspected that LRBA might similarly be important in epithelia for the subcellular distribution of membrane receptors, transporters or channel proteins. We therefore tested for a number of transport functions for solutes in the stomach and small intestine, and for taste perception, targeting organs in which we observed LRBA expression. However, we could not detect anomalies of any of the parameters which we addressed. It is possible that we did not select the right parameters in the organs which we functionally investigated, or that the respective cell types possess mechanisms which can compensate for LRBA deficiency. As a first epithelial dysfunction caused by LRBA deficiency, the perturbed trafficking of aquaporin-2 (AQP2) in the inner medullary collecting ducts of the kidney was recently determined^25^.

LRBA and NBEA are two closely related members within the BEACH protein family. As they are co-expressed in neurons and endocrine cells, we compared their subcellular distributions in cerebellar Purkinje cells and adrenal chromaffin cells by immuno-EM. In Purkinje cells, NBEA immunolabel concentrates markedly at the concave (putative *trans*-) sides of Golgi stacks, mainly associated with larger tubulo-cisternal endomembrane profiles, but is scarcer in the rest of the cytoplasm. In contrast, LRBA immunolabel is scattered throughout the Purkinje cell cytoplasm, occasionally associated with endomembranes, but not at all enriched near Golgi stacks. Chromaffin cells display a similar, largely complementary distribution of the two proteins, though not as strikingly as Purkinje cells. In chromaffin cells, LRBA also decorates the *trans*-Golgi region, though less markedly than NBEA. We consider this complementary subcellular localization of NBEA and LRBA to be a morphological correlate of complementary cell-biological functions, involving distinct cargo proteins and transport vesicle populations.

In chromaffin cells, we additionally detected NBEA immunoreactivity in the lumen of a subset of lysosome-like organelles, and also in association with aggregates of small vesicles and diffuse electron-dense material reminiscent of autophagy nucleation sites. Links between several BEACH proteins^26–28^, including LRBA^2,10,29^, and autophagy have been determined, and our EM observations suggest that also NBEA could be involved in autophagy, at least in endocrine cells. Autophagy is being intensely studied in insulin-secreting β cells (review^30^).

Human patients with *LRBA* mutations suffer from a plethora of pathologies due to immune deficiency (hypogammaglobulinemia, recurrent respiratory infections) or immune dysregulation (inflammatory bowel disorder, autoimmune enteropathies, haemocytopenias, or endocrinopathies). Most *LRBA*-mutant patients suffer from enteropathies, and endocrinopathies are also often observed (e.g. insulin-dependent diabetes mellitus, thyreoiditis, Addison disease), while manifestations such as hepatitis, arthritis, gastritis, end-stage renal disease, and cutaneous or neurological disorders are rare (Ref.^3^; and references therein). These manifestations are primarily attributed to immune dysfunctions, which is also supported by findings in LRBA-KO mouse models^5,6^. However, LRBA deficiency, even though it may not directly cause a clinically manifest gastrointestinal or endocrine pathology by itself, may render LRBA-expressing epithelial or endocrine cells more vulnerable to autoimmune attack or other forms of distress, or promote autoimmunity by autoantigen leakage, and thus contribute to the pathogenesis.

It has been observed in cell culture experiments that in β-cell-like MIN6 cells, LRBA knockdown reduced the storage and secretion of insulin^31^, suggesting also a direct LRBA deficiency phenotype in endocrine cells. However, intraperitoneal glucose tolerance tests, performed on our LRBA-KO mice by the German Mouse Clinic, did not detect an impairment of blood glucose clearance (results not shown). Also our IF results from pancreatic tissue in Fig. 3 gave no indication that the abundance or subcellular distribution of insulin in islets of Langerhans is affected by the LRBA-KO.

Given the high expression of LRBA in gastrointestinal epithelia, as well as its concentration toward the apical pole of small intestinal enterocytes, we were surprised that the detailed study of complex intestinal transport functions did not show significant differences between the LRBA-KO and WT mice. These transport functions involve the vesicle-associated trafficking of the respective transporter proteins to the apical membrane, and also the neural regulation of response to potentially noxious influences, like contact with luminal acid or hypotonicity. These negative results also indicate that the LRBA-KO mice did not suffer any marked acute or chronic illness, because in our experience virtually any disease (unless very organ-specific and not affecting the systemic well-being) affects the intricate regulation of the intestinal transport processes. Our LRBA-KO mice (official allele designation, *Lrba*^*tm1.1Kili*^) and also independent LRBA-KO mouse lines have been found to be healthy, with normal Mendelian ratios of homozygote, heterozygote and WT pups, normal developmental parameters and fertility, but a mild renal water loss that is compensated by polydipsy (Ref.^25^, Supplementary Fig. S3c–e, and our own unpublished data), and an increased susceptibility to dextran sulphate sodium (DSS)-induced colitis^5,6^. The latter studies were performed, because a prominent feature of *LRBA* mutations in humans is a severe, early-onset gastrointestinal phenotype with chronic diarrhea and colitis^32,33^. The DSS colitis model is an often-employed animal model to test for an enhanced susceptibility of a genetically altered mouse strain to intestinal damage, but produces very different damage severities depending on the age, genetic background, housing conditions, type and concentration of DSS, etc.^34^. The increased susceptibility of the LRBA-deficient mice to DSS colitis was explained by a defect on the regulatory T cells with a decreased CTLA-4 receptor expression^6^, which is a defect that also plays a prominent role in the human primary deficiency syndrome associated with increased autoimmunity and colitis^35^. Another study suggested a causal relationship between the increased susceptibility to DSS and an increased endosomal signaling of Toll-like receptors in LRBA-KO mice^5^. Toll-like receptors are indeed prominently expressed by all segments of the gastrointestinal tract, and are one of the links between the epithelia and the native immune system. Further studies into the prominent expression of LRBA in intestinal epithelia and the reduced immunological barrier function seem warranted.

## Materials and methods

### Gene-modified mice

Hypomorphic gene-trap mice (laboratory code, *Lrba/gt;* official allele designation, *Lrba*^*Gt(XE315)Byg*^; MGI:4124772) were generated by blastocyst injection of the gene-trap ES cell line XE315 from BayGenomics (San Francisco, USA); the gene-trap was found to be inserted after exon 38 of the mouse *Lrba* gene. Null-mutant LRBA-KO mice (laboratory code, *Lrba2*; official allele designation, *Lrba*^*tm1.1Kili*^, MGI:5796558) were generated at TaconicArtemis (Köln, Germany) through homologous recombination and subsequent in-vivo Cre deletion of exon 4 of the mouse *Lrba* gene, causing a frame-shift. Both mouse lines are described in detail in Ref.^7^, and have been deposited with the European Mouse Mutant Archive (EMMA) (www.infrafrontier.eu/emma) under IDs EM:14925 and EM:14924, respectively. Experiments involving animals were performed in accordance with the respective national regulations of Sweden or Germany, and in compliance with the ARRIVE guidelines. Experimentation permits are specified below in the respective Methods paragraphs.

### Antibodies

Rabbit anti-LRBA (affinity-purified sera LRBA-39.2 and -42.1) has been described in Ref.^7^, and rabbit anti-NBEA in Ref.^12^. The following commercial antibodies for marker proteins were used. Anti-Na^+^K^+^-ATPase α1 subunit (anti-ATP1A1): Developmental Studies Hybridoma Bank a6f (mouse monoclonal). Anti-Insulin: Sigma-Aldrich 05-1108 (mouse monoclonal). Anti-Glucagon: Sigma-Aldrich G2654 (mouse monoclonal). Rab27 (E8): Santa Cruz sc-74586 (mouse monoclonal). α-Gustducin: Santa Cruz sc-395 (rabbit). EAAT1: Santa Cruz sc-7758 (goat). PLCβ2: Santa Cruz sc-31757 (goat). DDC: Santa Cruz sc-46909 (goat).

### LacZ histochemistry

The animals were fixed by perfusion with 0.2% glutaraldehyde in PBS supplemented by 100 mM MgCl_2_ and 50 mM EGTA. The organs, except for the brain, were then isolated and soaked in 10% sucrose in PBS for 2 h, 20% sucrose for 3 h, and 30% sucrose overnight. These samples were sliced to a thickness of 20 µm or less using a Leica CM3050 S cryostat, placed on glass coverslips, and allowed to dry. The brain was sliced to a thickness of 20–50 µm using a Leica VT1200 vibratome and stored in PBS. The samples were stained by immersion in LacZ solution A (100 mM MgCl_2_ and 50 mM EGTA in PBS) for 30 min, then in LacZ solution B (2 mM MgCl_2_, 0.01% Na-deoxycholate, and 0.02% NP-40 in PBS) for two hours, and finally in LacZ solution C (solution B with 5 mM K-ferricyanide, 5 mM K-ferrocyanide, and 0.5 mg/ml X-gal added) for 2–7 days. They were then washed with PBS. Cryostat sections were counterstained with Nuclear Fast Red, washed with water, dried, and mounted onto slides using DPX mounting medium. Vibratome sections were cleared in an increasing sucrose concentration (15% sucrose for 1 h, 30% for 2 h, 45% overnight, and 60% for an additional 24 h) in PBS with 2% Triton, mounted in 60% sucrose in PBS, and sealed with nail polish. All samples were visualized using a Nikon Eclipse Ti2 microscope equipped with a Nikon color camera.

### Immunofluorescence microscopy of pancreas, salivary glands and intestines

4%-PFA immersion-fixed organ specimens from *Lrba*^+*/*+^ and *Lrba*^*−/−*^ mice were cut into small blocks, cryoprotected in 15% and 30% sucrose (all in PBS) and frozen in liquid N_2_. Double immunofluorescence was performed on 10 µm cryosections, as described in Ref.^36^ with minor modifications. Cryosections were rehydrated in PBS, treated with 1% sodium dodecyl sulfate (SDS) for 5 min, blocked with 1% bovine serum albumin (BSA)/PBS for 15 min, and incubated with rabbit anti-LRBA (at dilution 1:1000 in PBS) together with mouse monoclonal primary antibodies against either anti-Na^+^/K^+^-ATPase (1:200), anti-insulin (1:5000), anti-glucagon (1:5000), or anti-Rab27 (1:100), overnight at 4 °C. After washing with PBS, slides were incubated with donkey anti-rabbit IgG Alexa Fluor 594 (Jackson ImmunoResearch cat. # 711-585-152, 1:400) and donkey anti-mouse IgG Alexa Fluor 488 (Jackson ImmunoResearch cat. # 715-545-151, 1:400) for 1 h at RT. Slides were washed with PBS and mounted with Fluoromount-G (Southern Biotech; cat. # 0100-20) for nuclear staining. Slides were viewed using a TCS SP8 confocal laser scanning microscope with Plan-Apochromat CS2 20×/0.75NA IMM or CS2 40×/1.30NA oil immersion objective (Leica). Images were captured and analysed using the Leica Application System X (Las X) software. Both anti-LRBA-39.2 and -42.1 were employed for IF, yielding the same staining patterns.

### Pre-embedding immuno-EM

Immuno-EM with anti-LRBA-39.2 as primary antibody and nanogold-conjugated Fab’ as secondary antibody followed by silver enhancement was performed as in Ref.^37^.

### Immunofluorescence microscopy of taste buds, gustatory testing

Double-IF, including double-IF employing two primary antibodies from the same host species (rabbit anti-LRBA and anti-α-Gustducin), and co-localization analysis were performed as in Refs.^15,38^. Lickometer measurements on LRBA-KO male and female mice and littermate WT controls were performed as in Ref.^38^, Supplementary Fig. S3a,b and Supplementary Table S1. Lickometer protocols were adjusted to the water loss caused by the renal LRBA-KO phenotype^25^, which is compensated by polydipsy (increased drinking) of the KO mice (Supplementary Fig. 3c–e). Experiments were carried out under Animal Experimentation Permit number 2347-41-2014, issued by Landesamt für Umwelt, Gesundheit und Verbraucherschutz (Land Brandenburg).

### Duodenal epithelium transport functions

The surgical procedure and duodenal preparation were performed according to previously described procedures^39,40^. In brief, the mice were anesthetized by spontaneous inhalation of isoflurane and a catheter was placed in the left carotid artery to monitor blood pressure and for continuous infusion of an isotonic sodium carbonate solution (200 mM Na^+^ and 100 mM CO_3_^2−^) at a rate of 0.35 ml·h^−1^, to maintain normal acid–base balance^39^. A laparotomy was performed and a silicone tube was introduced through a hole made in the fore-stomach, guided through the stomach and pylorus and secured by a ligature 2–3 mm distal to the pylorus. A cannula was inserted into the duodenum ~ 1.5 cm distal to the pylorus and secured by ligatures. The duodenal proximal tubing was connected to a peristaltic pump and the segment was perfused with isotonic saline (154 mM NaCl) at a rate of 0.25 ml·min^−1^, and the perfusate was collected in 10-min samples. ^51^Cr-EDTA was administered intravenously as a bolus of ~ 75 µCi followed by a continuous infusion at a rate of ~ 50 µCi per hour. The rate of luminal alkalinization was determined by back-titration of the perfusate to pH 4.90 with 10 mM HCl under continuous gassing (100% N_2_) by using pH–stat equipment and is expressed as micromoles of base secreted per centimeter of intestine per hour (µmol·cm^−1^·h^−1^). The luminal perfusate and blood plasma were analysed for ^51^Cr-activity using a gamma counter (1282 Compugamma CS, Pharmacia, Uppsala, Sweden). The clearance of ^51^Cr-EDTA from the blood to the lumen was calculated as described^40^ and is expressed as (ml·min^−1^·100 g^−1^). The net fluid flux across the duodenal mucosa was determined by subtracting the perfusate volume per 10 min from the peristaltic pump volume per 10 min and is expressed as ml fluid per gram wet tissue weight per hour (ml·g^−1^·h^−1^). Descriptive statistics are expressed as the mean ± SEM, with the number of experiments given in parentheses. The statistical significance of the functional data was tested by repeated measures analysis of variance. To test the differences within a group, a 1-factor repeated measures ANOVA was used followed by a Tukey post-hoc test. Between groups, a 2-way repeated measures ANOVA was used followed by a Bonferroni post-hoc test. All statistical analyses were performed on an IBM-compatible computer using GraphPad Prism 5.03 software. P values less than 0.05 were considered significant. All experiments were performed according to the Guide for the Care and Use of Laboratory Animals of the National Institutes of Health and approved by Uppsala Ethics Committee for Experiments with Animals (Permit number: C121/8).

### Gastric and intestinal transport functions

(a) Basal and acid-stimulated duodenal bicarbonate secretory rates in vivo*:* LRBA-KO and WT littermates were anesthetized using isoflurane, the abdomen was opened and a duodenal loop was cannulated as described^41^. The brief exposure of the duodenal mucosa to a low pH (pH 2.5, 5 min) and the subsequent experimental procedure was performed as described^42^. The duodenal outflow was continuously collected and pH–stat titration was performed in 10 min portions to determine the bicarbonate concentration. (b) Gastric acid secretion in vitro: The acid secretory rate of LRBA-KO and WT littermates were determined in microdissected isolated gastric mucosa from the fundus/corpus area by pH–stat microtitration of the luminal perfusate in Ussing-chamber setups, as previously described^43^. In brief, the mucosal layer was dissected from the mouse gastric corpus under a stereomicroscope and mounted between two Lucite half chambers of a water-jacketed Ussing system equipped with a gas-lift system, as described^44^ with minor modifications as described^45^ and a different pH–stat/voltage current clamp (VCC) system (pH–stat titration systems by Hach Lange GmbH, Düsseldorf, Germany; custom-made digital VCC system and software, Klaus Mussler, Aachen, Germany). Maximal acid secretory capacity was investigated by serosal application of 10 μM forskolin (FSK) and 100 μM IBMX. (c) For the determination of jejunal fluid absorptive rate, the experiments were performed as described^20^, with minor modifications^46^. The intestinal segment was sequentially perfused at a rate of 30 ml h^−1^, first with an unbuffered solution, pH titrated to 7.4, 37 °C, consisting of 145.5 mM NaCl, 4.0 mM KCl, 1.2 mM CaCl_2_; then a 5%CO_2_/95%O_2_-gassed, HCO_3_^−^ buffered perfusate (121 mM NaCl; 24 mM NaHCO_3_; 4.0 mM KCl; 1.2 mM CaCl_2_); and finally a glucose-containing 5%CO_2_/95%O_2_-gassed, HCO_3_^−^ buffered perfusate (25 mM glucose, 96 mM NaCl; 24 mM NaHCO_3_; 4.0 mM KCl; 1.2 mM CaCl_2_). The rate of fluid absorption was calculated according to the weight of the influx and the outflow (effluent). Measurements were performed under Animal Experimentation Permit number 33.9-42502-04-11/0496 issued by Niedersächsisches Landesamt für Verbraucherschutz und Lebensmittelsicherheit.

### Supplementary Information

Supplementary Information.

## Data Availability

The data generated in this study are available from the corresponding author upon reasonable request.

## References

[1] Cullinane AR, Schäffer AA, Huizing M (2013). The BEACH is hot: A LYST of emerging roles for BEACH-domain containing proteins in human disease. Traffic.

[2] Lopez-Herrera G (2012). Deleterious mutations in LRBA are associated with a syndrome of immune deficiency and autoimmunity. Am. J. Hum. Genet..

[3] Jamee M (2021). Comprehensive comparison between 222 CTLA-4 haploinsufficiency and 212 LRBA deficiency patients: A systematic review. Clin. Exp. Immunol..

[4] Lo B (2015). Patients with LRBA deficiency show CTLA4 loss and immune dysregulation responsive to abatacept therapy. Science.

[5] Wang KW (2019). Enhanced susceptibility to chemically induced colitis caused by excessive endosomal TLR signaling in LRBA-deficient mice. Proc. Natl. Acad. Sci. U.S.A..

[6] Sudan R (2022). LRBA deficiency can lead to lethal colitis that is diminished by SHIP1 agonism. Front. Immunol..

[7] Kurtenbach S (2017). The BEACH protein LRBA promotes the localization of the heterotrimeric G-protein G_olf_ to olfactory cilia. Sci. Rep..

[8] Park MY (2016). LRBA is essential for allogeneic responses in bone marrow transplantation. Sci. Rep..

[9] Burnett DL, Parish IA, Masle-Farquhar E, Brink R, Goodnow CC (2017). Murine LRBA deficiency causes CTLA-4 deficiency in Tregs without progression to immune dysregulation. Immunol. Cell Biol..

[10] Gámez-Díaz L (2017). Immunological phenotype of the murine Lrba knockout. Immunol. Cell Biol..

[11] Vogl C (2017). The BEACH protein LRBA is required for hair bundle maintenance in cochlear hair cells and for hearing. EMBO Rep..

[12] Wang X (2000). Neurobeachin: A protein kinase A-anchoring, beige/Chediak-higashi protein homolog implicated in neuronal membrane traffic. J. Neurosci..

[13] Roper SD, Chaudhari N (2017). Taste buds: Cells, signals and synapses. Nat. Rev. Neurosci..

[14] Taruno A (2021). Taste transduction and channel synapses in taste buds. Pflügers Arch..

[15] Lossow K, Hermans-Borgmeyer I, Meyerhof W, Behrens M (2020). Segregated expression of ENaC subunits in taste cells. Chem. Sens..

[16] Takeuchi K, Matsumoto J, Ueshima K, Okabe S (1991). Role of capsaicin-sensitive afferent neurons in alkaline secretory response to luminal acid in the rat duodenum. Gastroenterology.

[17] Singh AK (2012). Neuronal cGMP kinase I is essential for stimulation of duodenal bicarbonate secretion by luminal acid. FASEB J..

[18] Seidler U (2008). Sodium and chloride absorptive defects in the small intestine in Slc26a6 null mice. Pflugers Arch..

[19] Seidler U (2009). The role of the NHERF family of PDZ scaffolding proteins in the regulation of salt and water transport. Ann. N. Y. Acad. Sci..

[20] Xia W (2014). The distinct roles of anion transporters Slc26a3 (DRA) and Slc26a6 (PAT-1) in fluid and electrolyte absorption in the murine small intestine. Pflugers Arch..

[21] Lin R (2011). D-glucose acts via sodium/glucose cotransporter 1 to increase NHE3 in mouse jejunal brush border by a Na+/H+ exchange regulatory factor 2-dependent process. Gastroenterology.

[22] Nair R (2013). Neurobeachin regulates neurotransmitter receptor trafficking to synapses. J. Cell Biol..

[23] Miller AC, Voelker LH, Shah AN, Moens CB (2015). Neurobeachin is required postsynaptically for electrical and chemical synapse formation. Curr. Biol..

[24] Gromova KV (2018). Neurobeachin and the kinesin KIF21B are critical for endocytic recycling of NMDA receptors and regulate social behavior. Cell Rep..

[25] Hara Y (2022). LRBA is essential for urinary concentration and body water homeostasis. Proc. Natl. Acad. Sci. U.S.A..

[26] Dragich JM (2016). Autophagy linked FYVE (Alfy/WDFY3) is required for establishing neuronal connectivity in the mammalian brain. Elife.

[27] Liu X (2017). The BEACH-containing protein WDR81 coordinates p62 and LC3C to promote aggrephagy. J. Cell Biol..

[28] Sim J (2019). The BEACH domain is critical for blue cheese function in a spatial and epistatic autophagy hierarchy. Front. Cell Dev. Biol..

[29] Nguyen TN (2021). ATG4 family proteins drive phagophore growth independently of the LC3/GABARAP lipidation system. Mol. Cell.

[30] Vivot K, Pasquier A, Goginashvili A, Ricci R (2020). Breaking bad and breaking good: β-cell autophagy pathways in diabetes. J. Mol. Biol..

[31] Hawari I (2022). Understanding the mechanism of diabetes mellitus in a LRBA-deficient patient. Biology.

[32] Kammermeier J (2017). Phenotypic and genotypic characterisation of inflammatory bowel disease presenting before the age of 2 years. J. Crohns Colitis.

[33] Tegtmeyer D, Seidl M, Gerner P, Baumann U, Klemann C (2017). Inflammatory bowel disease caused by primary immunodeficiencies—Clinical presentations, review of literature, and proposal of a rational diagnostic algorithm. Pediatr. Allergy Immunol..

[34] Katsandegwaza B, Horsnell W, Smith K (2022). Inflammatory bowel disease: A review of pre-clinical murine models of human disease. Int. J. Mol. Sci..

[35] Kardelen AD (2021). LRBA deficiency: A rare cause of type 1 diabetes, colitis, and severe immunodeficiency. Hormones.

[36] Brandes A (2007). Adaptive redistribution of NBCe1-A and NBCe1-B in rat kidney proximal tubule and striated ducts of salivary glands during acid-base disturbances. Am. J. Physiol. Regul. Integr. Comp. Physiol..

[37] Limbach C (2011). Molecular in situ topology of Aczonin/Piccolo and associated proteins at the mammalian neurotransmitter release site. Proc. Natl. Acad. Sci. U.S.A..

[38] Lossow K, Meyerhof W, Behrens M (2020). Sodium imbalance in mice results primarily in compensatory gene regulatory responses in kidney and colon, but not in taste tissue. Nutrients.

[39] Sjöblom M, Nylander O (2007). Isoflurane-induced acidosis depresses basal and PGE(2)-stimulated duodenal bicarbonate secretion in mice. Am. J. Physiol. Gastrointest. Liver Physiol..

[40] Wan Saudi WS, Sjöblom M (2017). Neuropeptide S reduces duodenal bicarbonate secretion and ethanol-induced increases in duodenal motility in rats. PLoS ONE.

[41] Singh AK (2010). The switch of intestinal Slc26 exchangers from anion absorptive to HCO_3_^−^ secretory mode is dependent on CFTR anion channel function. Am. J. Physiol. Cell Physiol..

[42] Singh AK (2013). Molecular transport machinery involved in orchestrating luminal acid-induced duodenal bicarbonate secretion in vivo. J. Physiol..

[43] Li T (2018). Genetic ablation of carbonic anhydrase IX disrupts gastric barrier function via claudin-18 downregulation and acid backflux. Acta Physiol..

[44] McDaniel N (2005). Role of Na–K–2Cl cotransporter-1 in gastric secretion of nonacidic fluid and pepsinogen. Am. J. Physiol. Gastrointest. Liver Physiol..

[45] Song P (2011). Kir4.1 channel expression is essential for parietal cell control of acid secretion. J. Biol. Chem..

[46] Tan Q (2021). Inhibition of Na+/H+ exchanger isoform 3 improves gut fluidity and alkalinity in cystic fibrosis transmembrane conductance regulator-deficient and F508del mutant mice. Br. J. Pharmacol..

