# Supplementary material for: LRBA, a BEACH protein mutated in human immune deficiency, is widely expressed in epithelia, exocrine and endocrine glands, and neurons
Source: Sci Rep. 2024 May 9;14:10678. doi: 10.1038/s41598-024-60257-6 (PMC11082223; doi:10.1038/s41598-024-60257-6)
Supplement: Supplementary file 1 — Supplementary Information. [file 41598_2024_60257_MOESM1_ESM.pdf]

Supplementary Material

Title:

LRBA, a BEACH protein mutated in human immune deficiency, is widely expressed in epithelia, exocrine and endocrine glands, and neurons

Authors:
Eleni Roussa, Pavel Juda, Michael Laue, Oliver Mai-Kolerus, Wolfgang Meyerhof, Markus Sjöblom, Katerina Nikolovska, Ursula Seidler & Manfred W. Kilimann

Supplementary Fig. S1:
LRBA cellular distribution in exocrine pancreas and submandibular gland

a

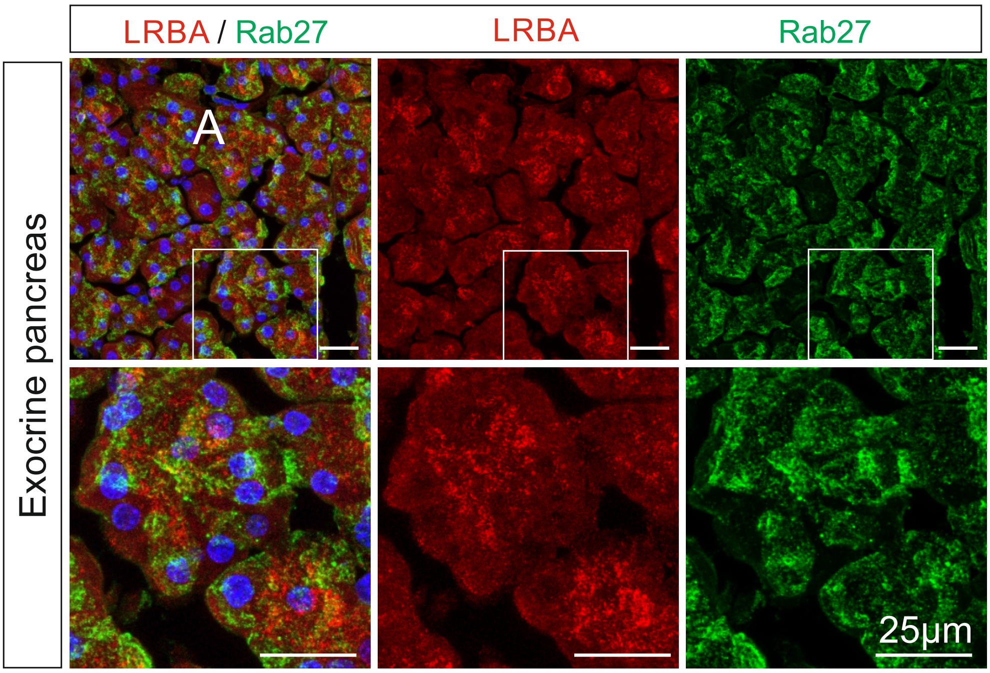

a) Double immunofluorescence for LRBA (red) and Rab27 (green) in acinar cells (A) of exocrine pancreas shows no colocalisation of the proteins. Nuclei were stained with DAPI. Lower row represents higher magnification images from the white-boxed areas.

b

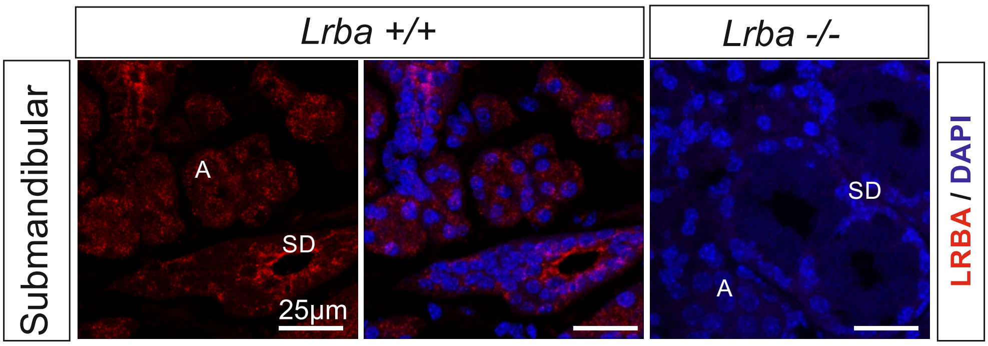

b) LRBA immunofluorescence (red) in apical and subapical areas of acinar (A) and striated duct (SD) cells was detected in submandibular gland of *Lrba*$^{+/+}$ but not of *Lrba*$^{-/-}$ mice. Nuclei were stained with DAPI.

Supplementary Fig. S2:

Different subcellular distributions of LRBA and NBEA, demonstrated by pre-embedding immuno electron microscopy of neuronal Purkinje cells of the cerebellum, and of endocrine chromaffin cells of the adrenal medulla.

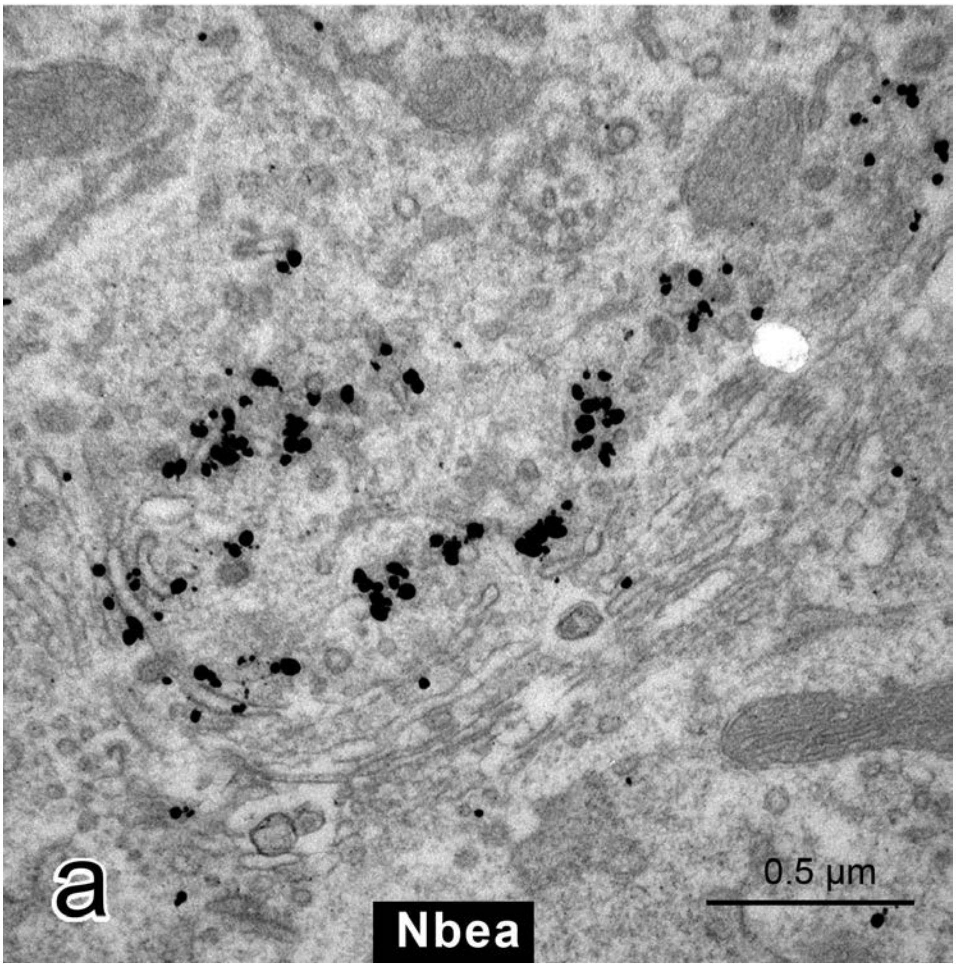

a) In Purkinje cells, NBEA immunolabel prominently decorates tubulo-cisternal endomembranes near the concave face and edges of Golgi stacks.

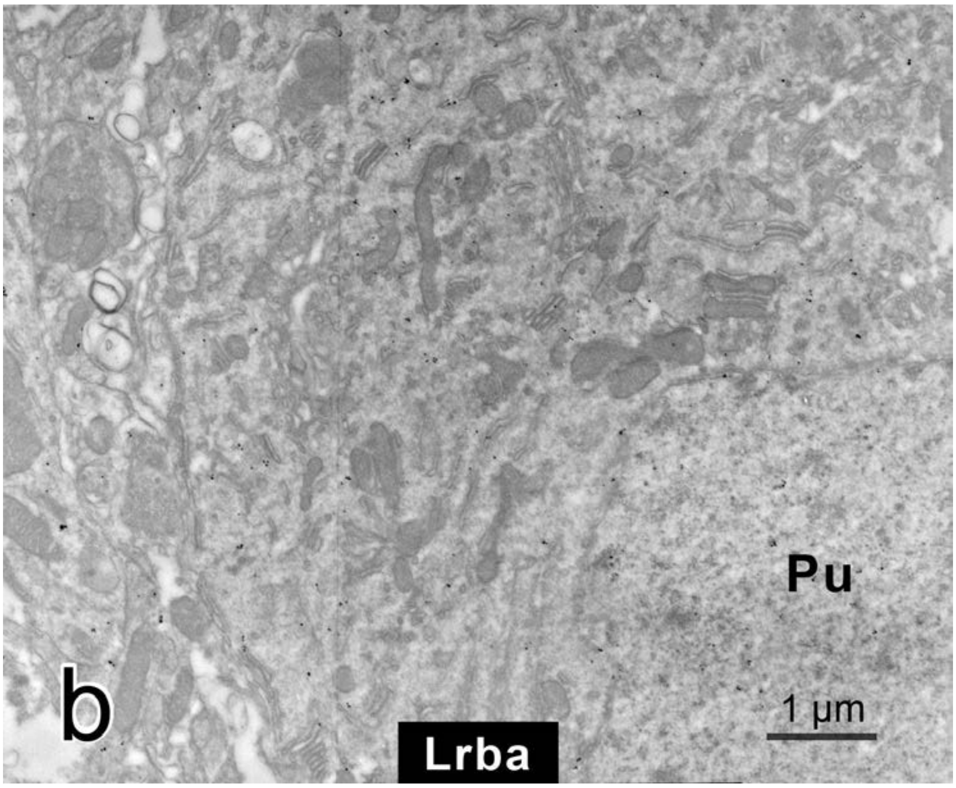

b) Knockout mouse specificity control of the LRBA antibody. In KO mouse cerebellum immunostained in parallel to the WT mouse tissue shown in part (c), Purkinje cell cytoplasm is essentially unlabeled.

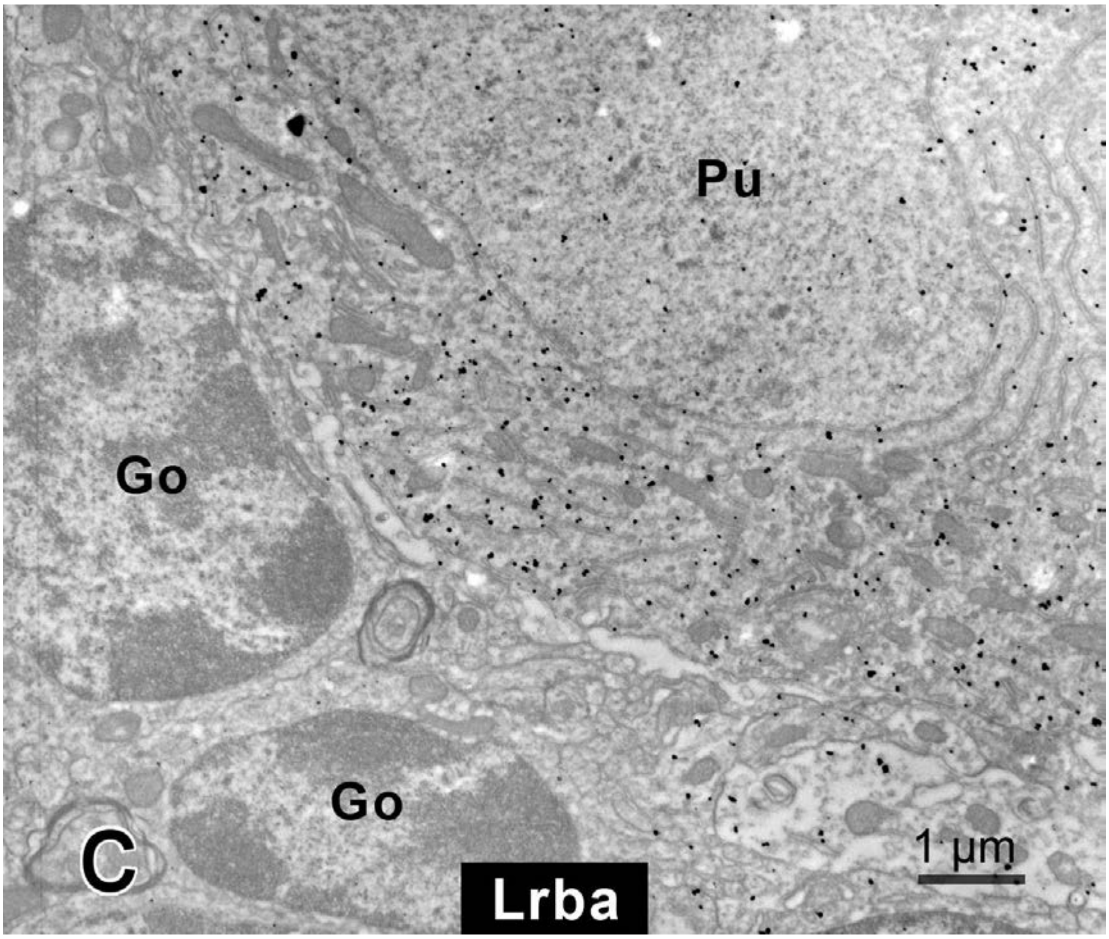

c) LRBA immunolabel is seen throughout the Purkinje cell (Pu) cytoplasm, whereas neighboring Golgi cells (Go) are unlabeled.

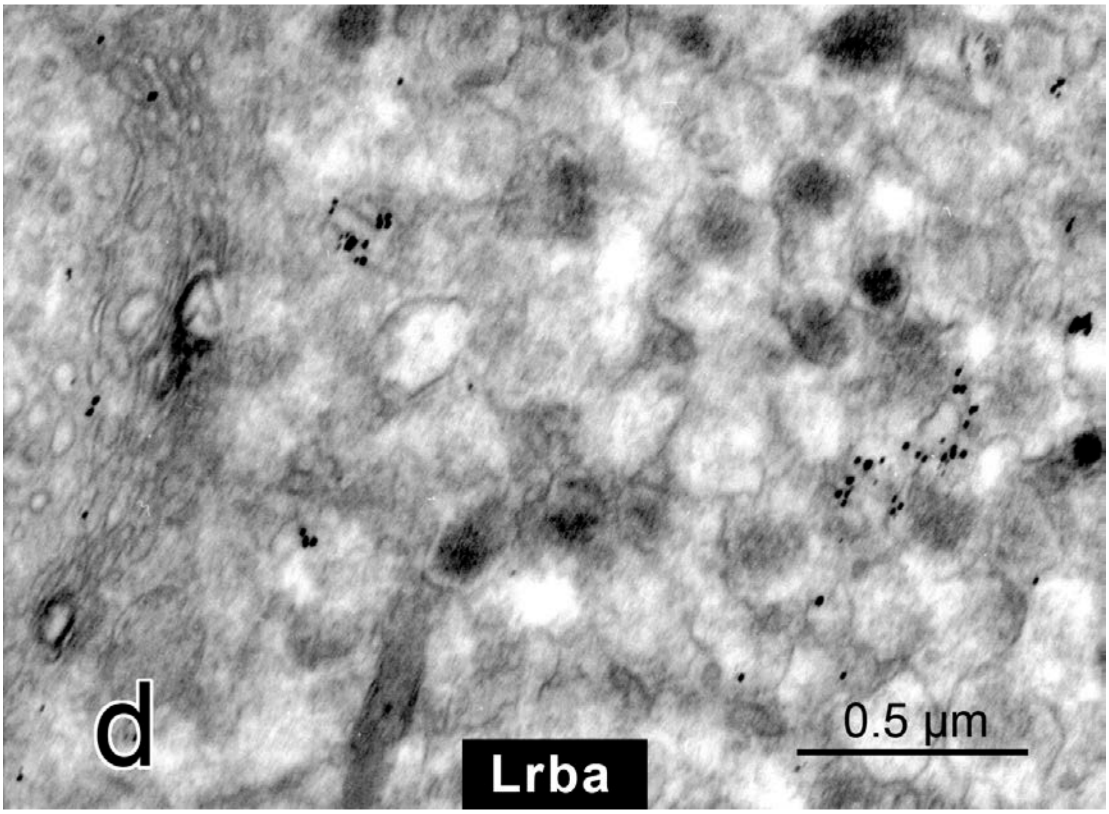

d) LRBA immunolabel in chromaffin cells is seen at the concave faces of Golgi stacks (left) as well as in association with polymorphic vesicles and vesicle clusters (center and right) throughout the cytoplasm.

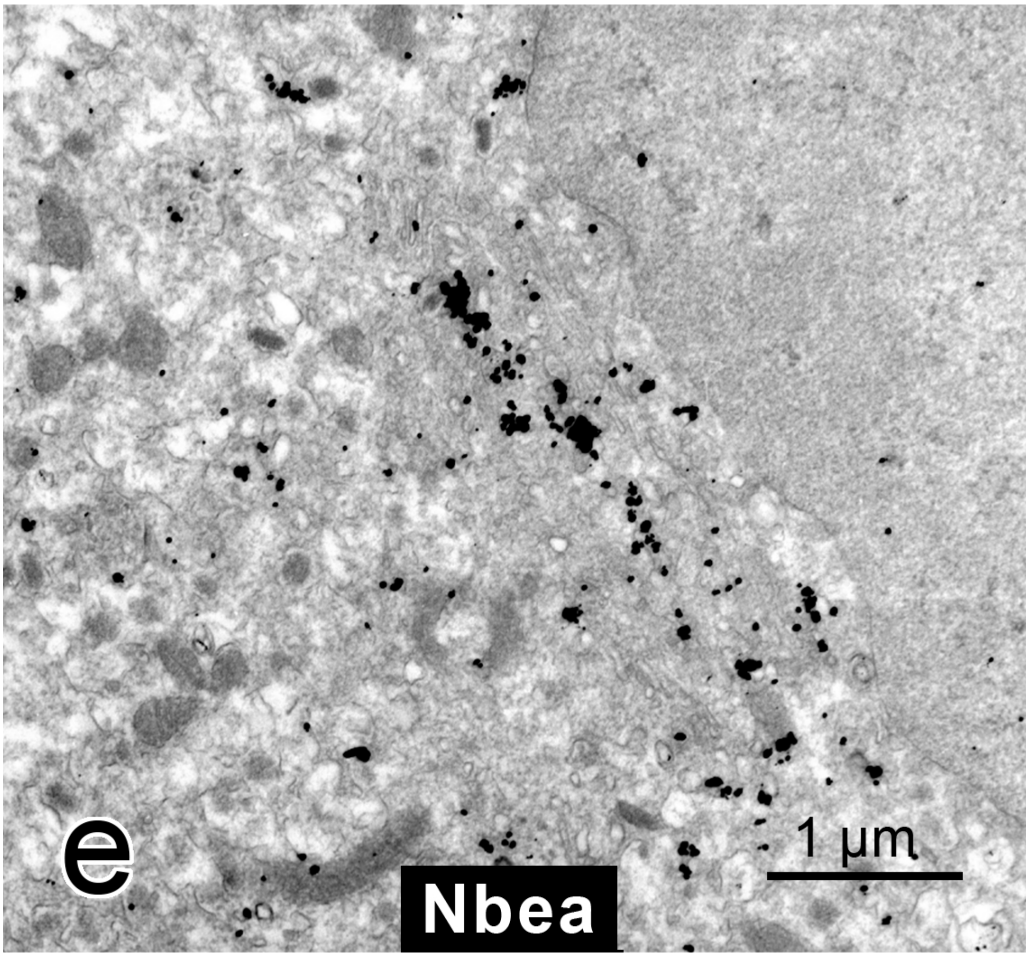

e) In chromaffin cells, NBEA immunolabel concentrates markedly at the concave faces and edges of Golgi stacks, similar to its localization in neurons (part a). Immunolabel over the rest of the cytoplasm is much sparser, but typically associated with tubulo-vesicular profiles or vesicle clusters.

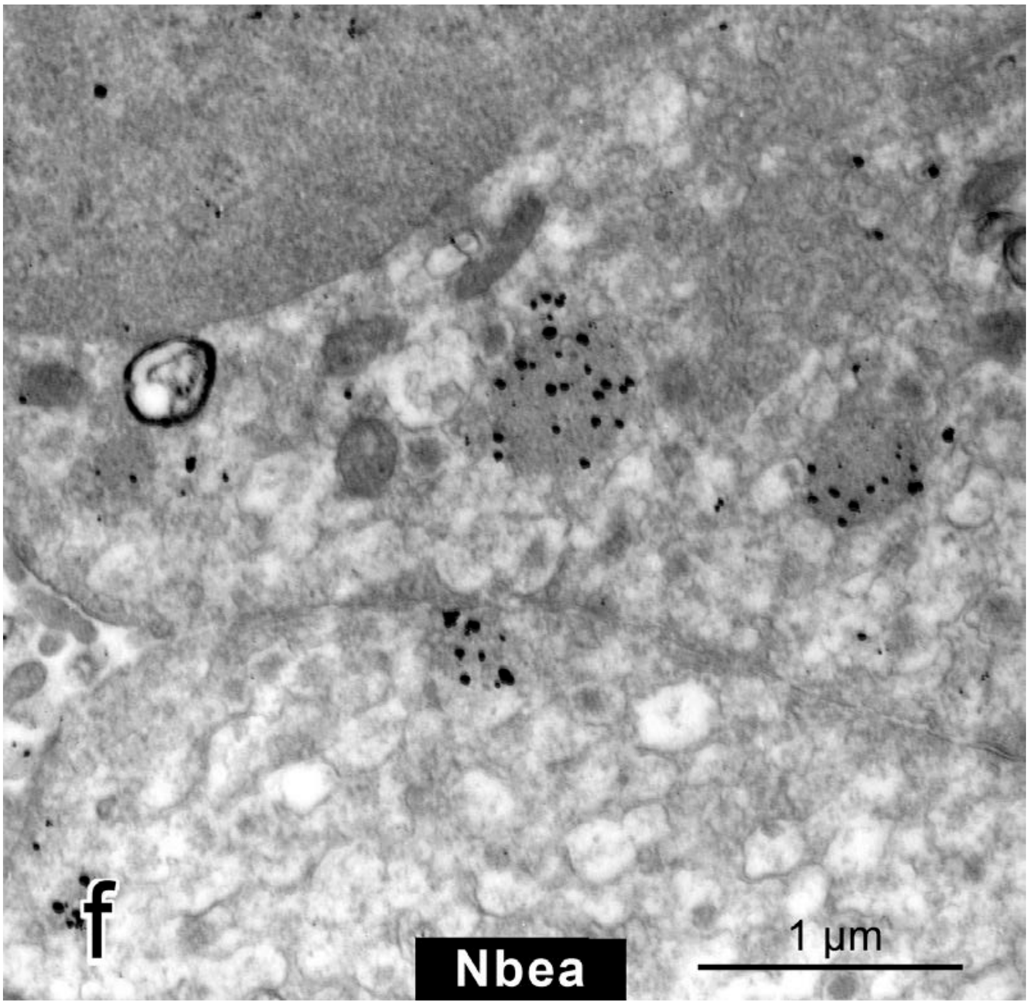

f) In chromaffin cells, NBEA immunolabel also decorates the lumen of a subpopulation of lysosome-like organelles (upper cell), as well as aggregates of small vesicles and diffuse electron-dense material (lower cell).

Supplementary Fig. S3: Gustatory measurements

a

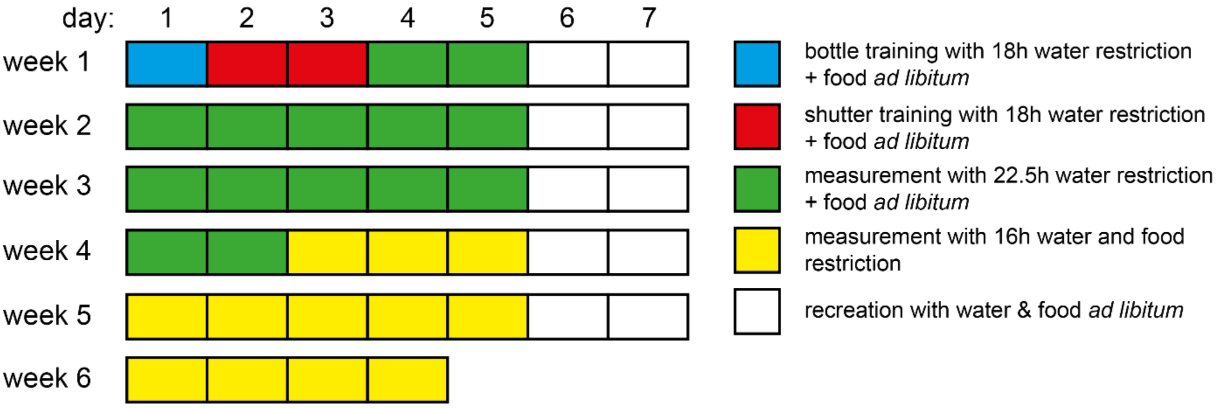

b

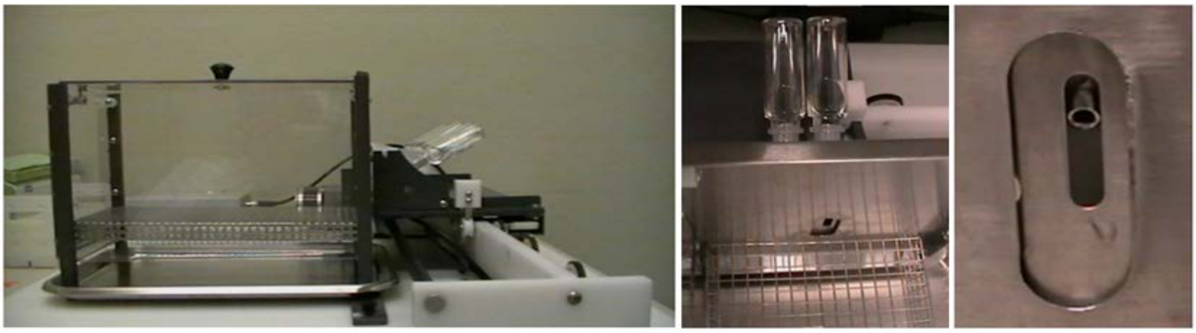

(a) Protocol and **(b)** setup (DavisRig lickometer) for taste preference tests.

Bottle training: 30min unlimited water access, shutter training: 30min water access 10s resp. 5s intervals, measurement: 20min access to test substances with 5s intervals

c

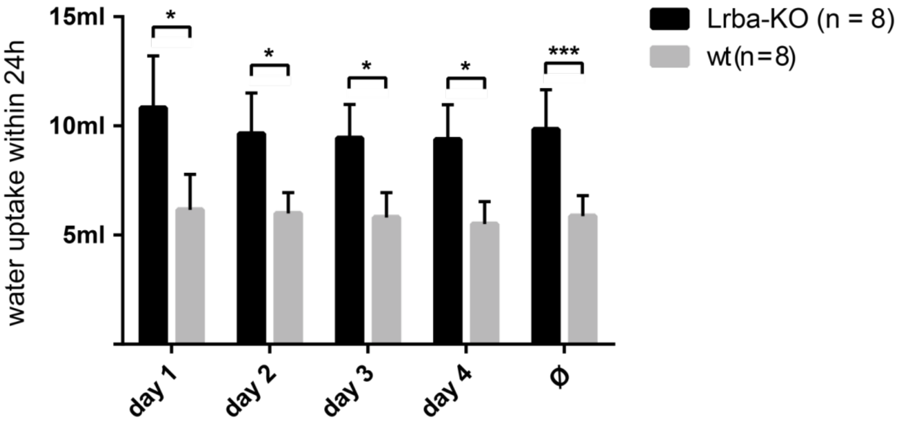

(c) LRBA-KO males drink 4 ml more water than their littermate controls in 24h.

(Two-way ANOVA with Sidak's multiple comparisons test; *, $p < 0.05$; ***, $p < 0.0001$)

d

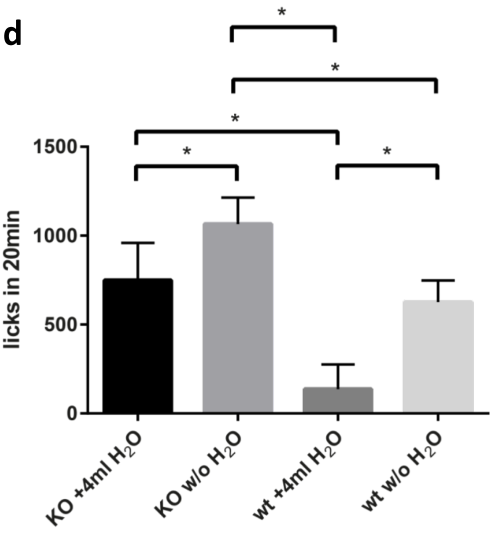

(d) Water-supplemented LRBA-KO males lick as often as unsupplemented littermate controls during aversive lickometer protocol (22.5h water restriction).

n = 4 animals, 4 repeats per animal. (Two-way ANOVA with Sidak‘s multiple comparisons test; *, $p < 0.05$)

e

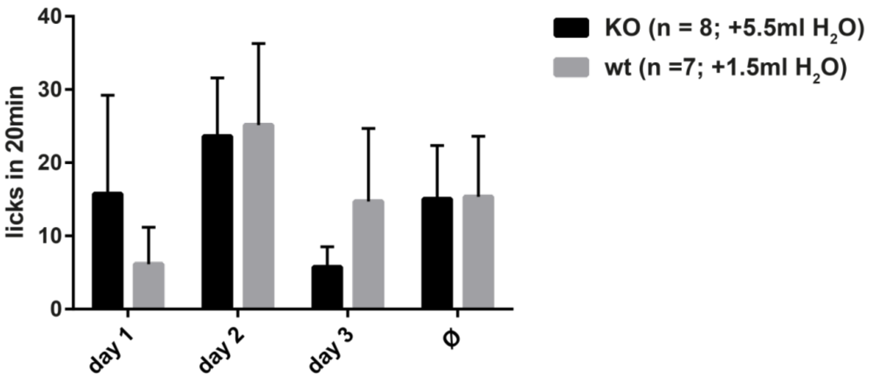

(e) Water-supplemented LRBA-KO males lick as often as unsupplemented littermate controls during attractive lickometer protocol (16h water restriction).

n = 8 animals, 3 repeats per animal. (Two-way ANOVA with Sidak‘s multiple comparisons test)

Supplementary Fig. S4: Duodenal epithelium transport functions

a

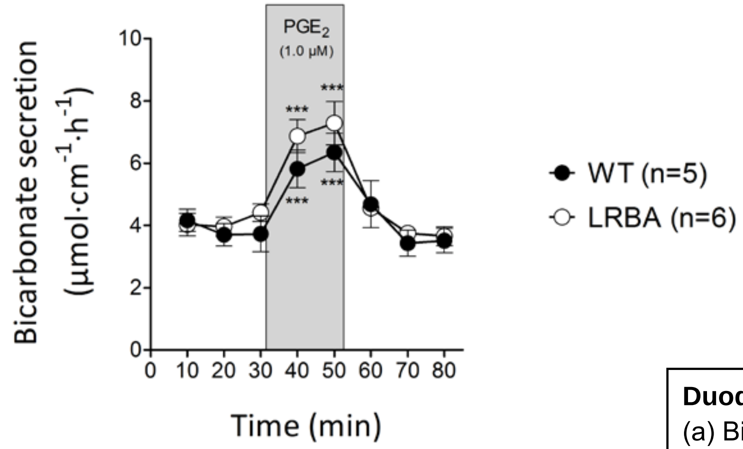

b

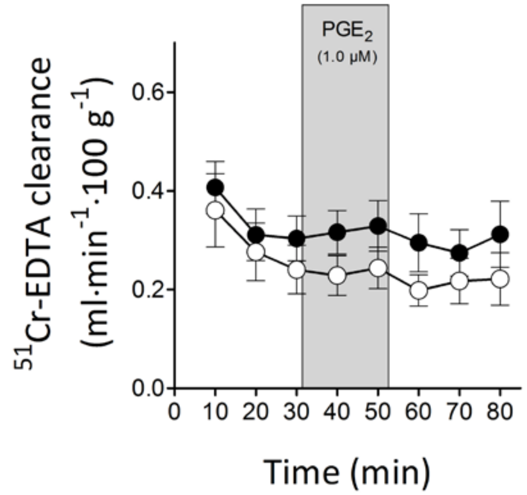

c

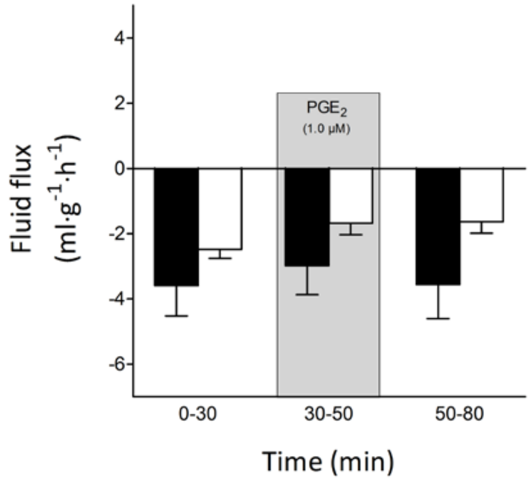

Duodenal mucosal barrier functions.
(a) Bicarbonate secretion
(basal & PGE_2-treated):
No genotype difference in basal bicarbonate secretion was observed, nor in the PGE_2 -stimulated secretory increase (WT from 3.7 ±0.6 to 6.4±0.6 µmol cm^{-1} h^{-1}; p< 0.001, n=5. LRBA-KO from 4.4±0.3 to 7.3±0.7 µmol cm^{-1} h^{-1}; p< 0.001, n=6).
(b) Paracellular permeability
(basal & PGE_2 -treated):
No genotype differences of blood-to-lumen clearance of ^{51}Cr-EDTA.
(c) Net fluid flux
(basal & PGE_2 -treated):
No genotype differences.
Values are mean ± SEM.
*** indicates significantly (P < 0.001) higher values compared to basal in the same group.

Supplementary Fig. S5: Gastric and intestinal transport functions

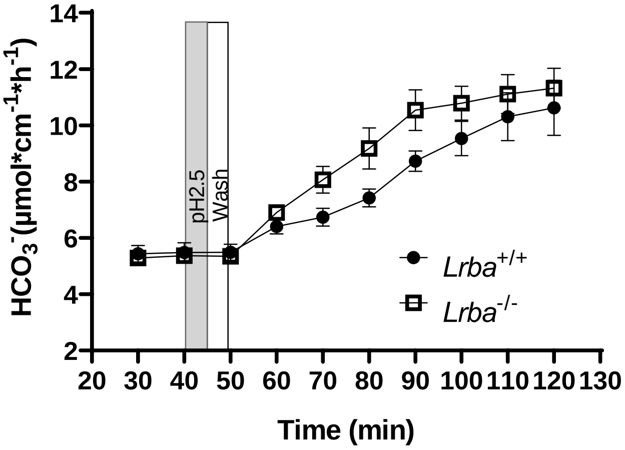

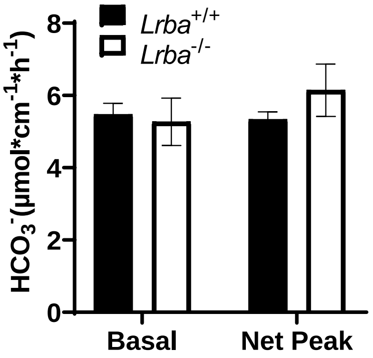

a: Basal and luminal-acid stimulated bicarbonate secretory rates in luminally perfused duodenum.
The alkaline output into the luminally perfused perfusate was determined by pH-stat microtitration in the duodenum of anesthetized LRBA-deficient and WT littermates. No significant difference in basal bicarbonate output rates or in the increase after a 5 min contact of the mucosa with a pH 2.5 was found between LRBA-deficient and WT mice. n=4/5 animals per genotype. Error bars: S.E.M.

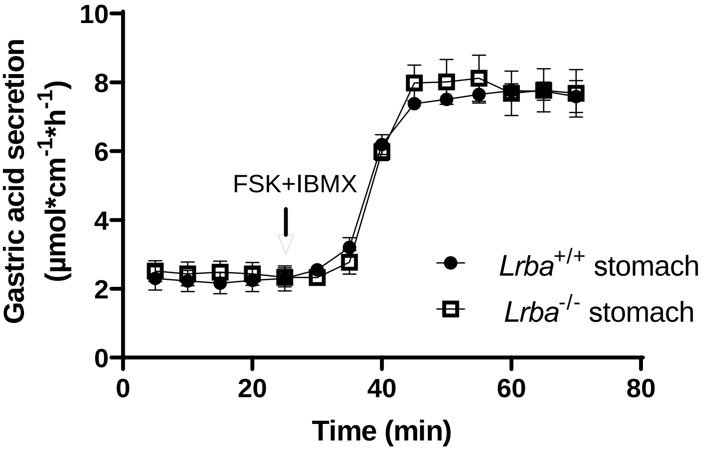

b: Basal and agonist-stimulated acid secretory rates in isolated gastric mucosa.
Isolated gastric epithelia from the corpus/fundus region, from which the muscle layers had been removed by microdissection, were mounted in Ussing-chamber systems and the basal and agonist-stimulated acid-secretory rate was determined by pH-stat titration of the luminal bath. The combination of forskolin (FSK, 10-5M) and the phosphodiesterase inhibitor IBMX (10-4M) generates maximal stimulation of acid secretory rate. No difference was observed in basal and stimulated acid secretory rate between LRBA-deficient and WT gastric epithelium. n=4.

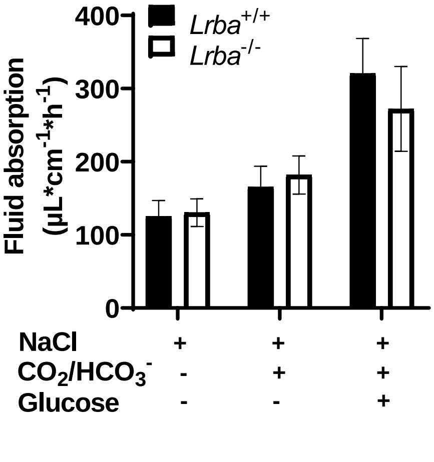

c: Basal, CO_2-stimulated and glucose-stimulated fluid absorptive rates in luminally perfused jejunum.
Fluid absorptive rates were determined gravimetrically after single-pass perfusion with saline, with a 5% CO_2 gassed/24mM HCO_3^- solution, or with saline containing 25 mM glucose, all at pH 7.4. Both the luminal CO_2 (via enterocyte entry, carbonic acid formation and stimulation of luminal Na^+/H^+ exchange) and luminal glucose (via stimulation of the Na^+/glucose cotransporter SGLT1) stimulated jejunal fluid absorptive rates. No significant difference was seen between LRBA-deficient and WT mice. n=5

Supplementary Table S1: Substances and their concentrations used in lickometer experiments.

The flavours “bitter (artificial)”, “bitter (natural)”, “salty” and "sour" were used with the lickometer protocol for aversive taste. “sweet (artificial)”, “sweet (natural)” and “umami” were used with the attractive taste protocol.

Flavour or substance	Lowest and highest concentrat. [mM] in lickometer measurements		Oral LD_{50} dose [mg kg^{-1}]
**bitter (artificial)**			
cycloheximide	0.001	0.1	133
denatonium benzoate	0.1	10	584 (rat)
**bitter (natural)**			
quinine	0.1	10	800
papaverin	0.1	10	162
**salty**			
potassium chloride	10	1000	1500
sodium chloride	10	1000	2,602 (i.p.)
**sour**			
hydrochloric acid	1	100	450 (i.p.)
citric acid	1	300	>5,000
**sweet (artificial)**			
aspartame	0.1	100	>5,000
sucralose	0.1	100	>16,000
**sweet (natural)**			
polycose	10	1000	>44,000
sucrose	10	1000	>16,000
**umami**			
mono sodium glutamate	3	1000	>12,000
+ inositol mono phosphate	-	1	10,000
+ amiloride	-	0,1	56
mono potassium glutamate	3	1000	4,500
+ inositol mono phosphate	-	1	10,000
+ amiloride	-	0,1	56
